# Atherton–Todd reaction: mechanism, scope and applications

**DOI:** 10.3762/bjoc.10.117

**Published:** 2014-05-21

**Authors:** Stéphanie S Le Corre, Mathieu Berchel, Hélène Couthon-Gourvès, Jean-Pierre Haelters, Paul-Alain Jaffrès

**Affiliations:** 1 Université de Brest, Université Européenne de Bretagne, CEMCA, CNRS UMR 6521, SynNanoVect, IFR 148 ScInBIoS, 6 Avenue Le Gorgeu, 29238 Brest, France

**Keywords:** amphiphiles, flame retardant, lipid conjugates, organophosphorus, phosphoramidate, phosphate

## Abstract

Initially, the Atherton–Todd (AT) reaction was applied for the synthesis of phosphoramidates by reacting dialkyl phosphite with a primary amine in the presence of carbon tetrachloride. These reaction conditions were subsequently modified with the aim to optimize them and the reaction was extended to different nucleophiles. The mechanism of this reaction led to controversial reports over the past years and is adequately discussed. We also present the scope of the AT reaction. Finally, we investigate the AT reaction by means of exemplary applications, which mainly concern three topics. First, we discuss the activation of a phenol group as a phosphate which allows for subsequent transformations such as cross coupling and reduction. Next, we examine the AT reaction applied to produce fire retardant compounds. In the last section, we investigate the use of the AT reaction for the production of compounds employed for biological applications. The selected examples to illustrate the applications of the Atherton–Todd reaction mainly cover the past 15 years.

## Review

### 1. Introduction

**Scheme 1:**
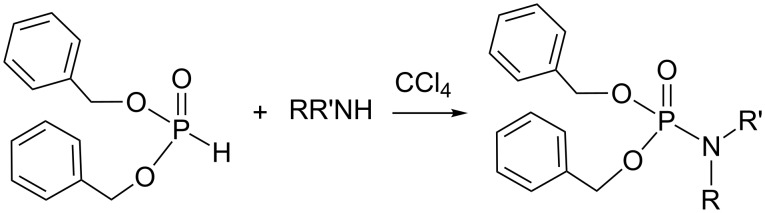
Pioneer works of Atherton, Openshaw and Todd reporting on the synthesis of phosphoramidate starting from dibenzylphosphite (adapted from [1]).

The reaction of dialkylphosphite with primary or secondary amines in the presence of a base in carbon tetrachloride produces phosphoramidates. This reaction, initially studied by F. R. Atherton, H. T. Openshawand, and A. R. Todd [1] in 1945 (Scheme 1) and now identified as the Atherton–Todd (AT) reaction, was actually discovered by chance. Indeed, these authors attempted to purify a solution of dibenzyl phosphite in carbon tetrachloride by its treatment with an aqueous ammonia solution. They observed the formation of a precipitate that was identified as *O*,*O*-dibenzyl phosphoramidate. However, no reaction occurred when dibenzyl phosphite was mixed alone with carbon tetrachloride, whereas an exothermal reaction occurred if gaseous ammonia was passed through this solution or if this solution was shaken with an aqueous ammonia solution, with chloroform and ammonium chloride as byproducts. The replacement of ammonia with primary and secondary amines yielded N-substituted phosphoramidates (Scheme 1). It is noteworthy, that less nucleophilic amines like aniline can also be engaged in the AT reaction, but the expected phosphoramidates are only produced in modest yields if a tertiary amine is added to the reaction media. These findings were published in the initial works of Atherton and Todd and completed in 1947 [2].

To the best of our knowledge, we herein report the first compilation of the works which studied or used this reaction. First the question of the mechanism of the Atherton–Todd reaction, which has been widely discussed in literature, is addressed. Then, the scope of this reaction is presented. Finally selected applications of this reaction are discussed. This selection mainly covers the applications of the past 15 years. A special focus is put on the synthesis of flame-retardant materials and the design of phosphorus-based amphiphilic compounds.

### 2. Mechanism of the Atherton–Todd reaction

**Scheme 2:**
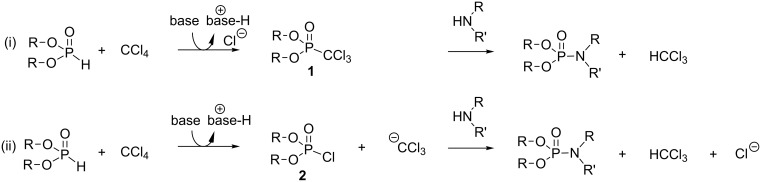
Mechanisms 1 (i) and 2 (ii) suggested by Atherton and Todd in 1945; adapted from [1].

In their initial publication, Atherton and Todd have suggested two possible mechanisms to explain the formation of phosphoramidate [1]. The first one (Scheme 2-i) was based on a two-step sequence with the formation of dialkyl trichloromethylphosphonate **1** as the intermediate species. The second mechanism, which was not preferred at that time by these authors (Scheme 2-ii), was based on the formation of dialkyl chlorophosphate **2** as a possible intermediate species. The preference for mechanism 1 was justified by the existence of some similitude with the reactivity of carbon tetrachloride reported in literature (e.g., the synthesis of arylcarboxylate according to a Reimer–Tiemann reaction [3–4]) and because the second step (i.e., the nucleophilic substitution on the electrophilic phosphorus) shares some characteristics with the reactivity of trichloroacetophenone (haloform reaction [5]). Moreover, the absence of the reactivity of alcohol when mixed with dialkyl phosphite and carbon tetrachloride in the presence of trialkylamine convinced the authors to prefer this first mechanism 1 (i). Nevertheless the same authors [2] revised their preference two years later and mentioned that the reaction may probably occur following mechanism 2 (Scheme 2-ii). This assumption was based on the observation that the replacement of carbon tetrachloride by bromotrichloromethane enhanced the reaction rate. They explained this increase of reactivity by the easier nucleophilic attack of the dialkylphosphite salt on the bromine atom of CBrCl_3_ when compared to the reaction with CCl_4_. It is also noteworthy, that the use of bromotrichloromethane allowed the phosphorylation of ethanol. The formation of tetrabenzyl pyrophosphate, observed by Atherton and Todd during the reaction that engaged benzyl phosphite, potassium hydroxide and carbon tetrachloride (or bromotrichloromethane), is also more easily explained by mechanism 2. Moreover, the impossibility to isolate the intermediate species in this reaction incited Atherton and Todd to prefer mechanism 2 (ii) because diethyl chlorophosphate **2** is a more reactive intermediate.

**Scheme 3:**
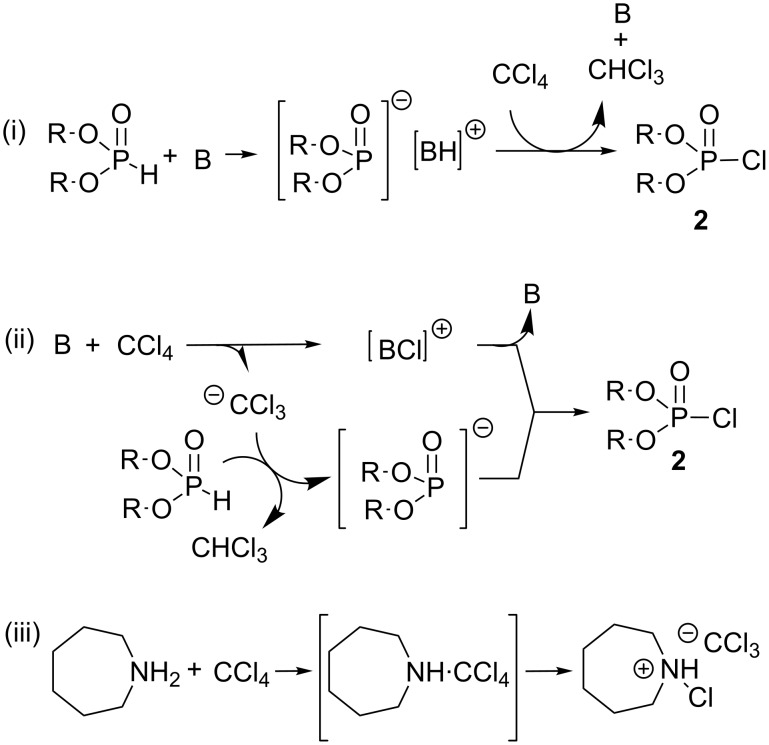
Two reaction pathways (i and ii) to produce chlorophosphate **2**. Charge-transfer complex observed when hexahydroazepine was used as a base (iii); adapted from [6] and [9].

After these pioneering works, the first investigation of the mechanism of the Atherton–Todd’s reaction was reported by Steinberg in 1950 [6]. In this work, the synthesis of dialkyl chlorophosphate **2** is reported by reacting dialkyl phosphite with carbon tetrachloride in the presence of 10 to 15 mol percent of trialkylamine acting as a catalyst. These authors proposed a more detailed mechanism for the formation of dialkyl chlorophosphate **2** with the suggestion of two distinct pathways (Scheme 3). One possibility included the nucleophilic attack of the deprotonated dialkyl phosphite on one chlorine atom of carbon tetrachloride (Scheme 3-i), whereas the second synthetic pathway (Scheme 3-ii) involved the nucleophilic attack of the base on carbon tetrachloride as a preliminary step. The kinetic data reported by Steinberg did not allow for discrimination between these two possibilities. Beside the kinetic study, Steinberg accumulated further interesting information relative to the mechanism of the Atherton–Todd reaction. First, Steinberg indicated that no reaction occurred when dialkyl trichloromethylphosphonate, prepared unambiguously by another method, was mixed with ammonia. This claim is in contradiction with one previous publication by Kamai [7–8], stipulating that phosphoramidate was produced by the reaction of dialkyl trichloromethylphosphonate and a selected amine. Steinberg has also shown that the structure of the amine has an influence on the rate of the reaction. Indeed, triethylamine is a much more efficient catalyst (1000 fold) than pyridine. Interestingly, tributylamine or tripentylamine catalyzed this reaction with the same rate than triethylamine. This last observation by Steinberg is in favor of the first mechanism (Scheme 3-i), since differences of the reaction rate should be expected for the nucleophilic addition of trialkylamine on carbon tetrachloride (Scheme 3-ii) depending on the structure of the alkyl chains. The works of Steinberg eliminated the possibility of a mechanism proceeding by radical processes. Indeed, the use of UV irradiation or radical initiators in the absence of trialkylamine was found to be unsuccessful to produce phosphoramidates. Recently, Krutikov et al. [9] have reported that hexahydroazepine (a secondary amine with a p*K*_a_ of 11.1 (Scheme 3-iii), used as a base in the AT reaction) probably reacted first with carbontetrachloride to produce a charge-transfer complex (this type of interaction was confirmed by refractometric titration). However, in this reaction the trichloromethylphosphonate, which would result from the reaction of the anion CCl_3_^−^ with chlorophosphate, was never observed because CCl_3_^−^ probably reacted as a base in the presence of dialkylphosphite (Scheme 3-ii). This experiment indicates that the basicity/nucleophilicity of the amine has an impact on the first step of the mechanism (a charge-transfer complex was not observed with a less basic amine like 2-aminopyridine) while the chlorophosphate **2** was assumed to be one common intermediate independent from the nature of the initial step.

Almost 35 years after the work of Steinberg, Engel et al. [10] have re-investigated the mechanism of the Atherton–Todd reaction, more specifically the first step (reaction of dialkyl phosphite with carbon tetrachloride and a base) by using CPG as an analytical tool. Triethylamine or sodium hydride was used as base. With sodium hydride the deprotonation occurs in a preliminary step to produce sodium dialkyl phosphite. All these attempts were never able to identify trace amounts of diethyl trichloromethylphosphonate (mechanism i, Scheme 2), thereby favoring mechanism 2 (Scheme 2). Additional experiments with the aim of discarding the involvement of a carbene species as an intermediate were carried out. The reactions achieved in cyclohexene (a solvent with the capacity to trap any traces of carbene) produced the same conversion rate. One exception to this last observation was found when the experimental conditions combined both the use of strict aprotic conditions (use of sodium hydride for the deprotonation of phosphite) and dimethyl phosphite as a substrate. This result might be rationalized by the instability of trichloromethanide in aprotic media which in the presence of dimethyl phosphite or dimethyl chlorophosphate produced a significant amount of carbene. It must be mentioned that methyl esters of phosphate or phosphonate have a particular reactivity since the methyl group can be easily removed by trimethylamine as illustrated by the dealkylation of *O*,*O*-dimethyl phosphoramidate [11–12] or by the thermal sensitivity of dimethyl chlorophosphate as mentioned by Steinberg et al. [6]. However, no trace amount of carbene was detected when triethylamine was replaced with sodium hydride as a base, while all other parameters were identical. These results indicated that the carbene pathway is unlikely to occur under the classical conditions of the Atherton–Todd reaction (dialkyl phosphite, trialkylamine, alkyl- or dialkylamine and carbon tetrachloride or bromotrichloromethane).

**Scheme 4:**
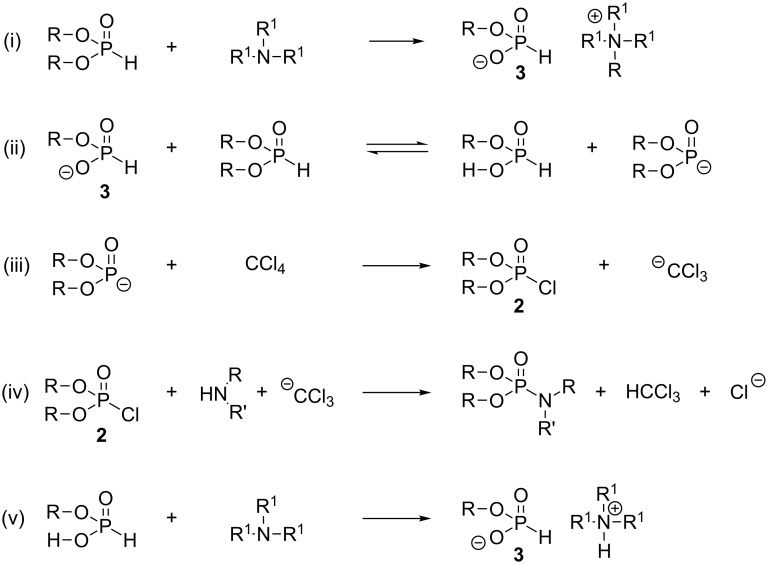
Mechanism of the Atherton–Todd reaction with dimethylphosphite according to Roundhill et al. (adapted from [13] and [17]).

The use of dimethyl phosphite as a substrate in the AT reaction was also studied by Roundhill and co-workers in a series of articles. They investigated the role of the salt **3** (Scheme 4-i), which can be produced by reacting trialkylamine with dialkyl phosphite [13]. Indeed, it was previously reported that this salt can catalyze (2 mol percent) an AT reaction [14–15]. However, there is no evidence that this mechanism could have a general scope. Roundhill et al. [16] have first studied the consequences of the replacement of carbon tetrachloride by a member of the chlorofluorocarbon class of compounds. In this study, they observed that the introduction of a fluorine atom in place of a chlorine atom reduced the reactivity leading to the following relative reactivity: CCl_4_ > CFCl _3_ > CF_2_Cl_2_ >> CHCl_3_. In this study, dimethyl phosphite was primarily used as a substrate, and cyclohexylamine as a nucleophilic amine. The formation of the salt **3** was observed when cyclohexylamine was added to dimethyl phosphite, thus pointing out the influence of the order of addition of the reactants.

Roundhill et al. [17] used computational chemistry to further investigate the mechanism of the Atherton–Todd reaction (HF-6.31G* level of theory and Moller–Plesset (MP2) to correct correlation effects). They found that the calculation supported the mechanism shown in Scheme 4 that starts with the dealkylation of dimethyl phosphite by an amine (Scheme 4-i). Then, this salt acted as a base to deprotonate dimethyl phosphite (Scheme 4-ii), which subsequently reacted with CCl_4_ to produce chlorophosphate **2** as an intermediate species. In the last step, the salt **3** is regenerated by deprotonation with trialkylamine (Scheme 4-v). Nevertheless, the scope of this theoretical study may be regarded as limited, since only dimethyl phosphite was considered in this study. It is indeed well-established that dimethyl phosphite has a singular reactivity when compared to others dialkyl phosphites [6,11]. Moreover, only unsubstituted amine (ammonia) and monosubstituted amine were considered in this theoretical study.

**Scheme 5:**
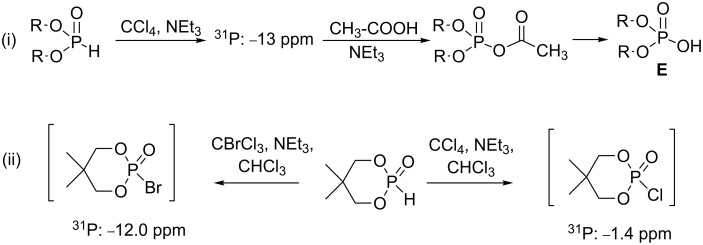
Synthesis of dialkyl phosphate from dialkyl phosphite (i) and identification of chloro- and bromophosphate as reaction intermediate (ii) (adapted from [18] (i) and [22] (ii)).

More recently, dialkyl trichloromethylphosphonate was again proposed as an intermediate species in a reaction producing dialkyl phosphate (Scheme 5-i) [18]. The authors postulated its formation on the basis of ^31^P NMR analysis. The ^31^P chemical shift reported for the intermediate in this study (−13 ppm) was not consistent with the chemical shift of dialkyl trichloromethylphosphonate reported in other studies (6.5 ppm [19–20]). A ^31^P chemical shift at −13 ppm would be more likely attributed to a pyrophosphate (−12.6 ppm, R = Et) [1], whereas diethyl chlorophosphate is observed at 3.5 ppm [21]. The formation of pyrophosphate could be explained by the presence of trace amounts of water concomitantly with the absence of nucleophile species. Then, the addition of acetic acid produced a mixed anhydride as suggested by the authors (Scheme 5-i). In another recent study, chloro- and bromophosphate intermediates were characterized by ^31^P NMR thus supporting the mechanism 2 (Scheme 2). In this study, Döring et al. reported that the shielding effect of bromine was also correlated to its higher reactivity (Scheme 5-ii) [22].

**Scheme 6:**
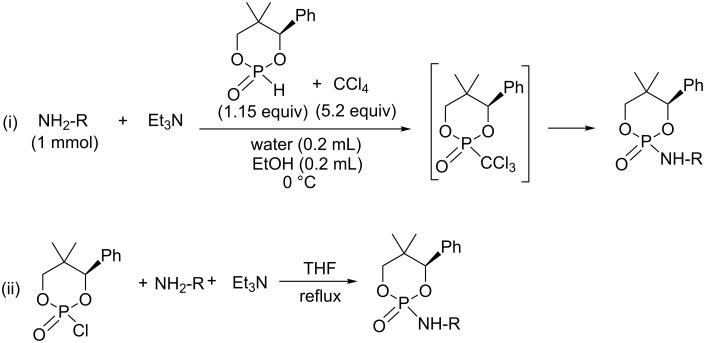
Synthesis of chiral phosphoramidate with trichloromethylphosphonate as the suggested intermediate (i) and directly from a chlorophosphate used as a substrate (ii) (adapted from [25–27]).

Feringa et al. developed a methodology for the determination of the enantiomeric excess of a chiral amine based on the use of phosphorinanes derivatives. Two synthetic pathways i) the AT reaction and ii) the direct use of chlorophosphate were evaluated for the derivatization of chiral amine (or alcohol) as reported in Scheme 6. For the AT reaction (i) [23] they used the conditions of Ji et al. [24], who have previously reported that the AT reaction can proceed in aqueous organic solvents (water/ethanol or water/DMF). Accordingly, a mixture of phosphorinane and CCl_4_ was added dropwise on a cooled (0 °C) solution of amino derivative and triethylamine in a water/ethanol mixture. For this reaction, the volume of the solvent mixture must be limited as mentioned in a review published by Feringa et al. [25]. The use of aqueous organic condition for the AT reaction led the authors to postulate trichloromethylphosphonate as an intermediate species because it is less sensitive to water when compared to chlorophosphate (Scheme 6) [26–27].

It must be noted that the intermediates were not characterized by NMR spectroscopy. Furthermore, we can hypothesize that when aqueous organic conditions are used, competitive reactions could take place on the chlorodioxaphosphorinane as an intermediate that would involve the different nucleophilic species present, which are water, ethanol and amine. Different experimental parameters listed below are in favor of the existence of competitive reactions that could have crucial consequences on the issue of the reaction and on the nature of the intermediate species: i) the addition of the phosphorinane and trialkylamine was achieved at 0 °C (this should favor the formation of the kinetic product). ii) Phosphorinane and trialkylamine were added dropwise on the primary amine (this is the best condition to have an excess of the nucleophilic amine versus the chlorophosphorinate intermediate and consequently to favor its addition on the chlorophosphate), iii) the low quantities of solvent used (this is also in favor of the addition of the nucleophilic amine, which is in competition with water and ethanol).

Moreover, these experimental details are also consistent with the reaction rate of chlorophosphate with nucleophiles reported by Corriu et al. [28]. Indeed, they have shown that the second-order rate constants for the solvolysis of diethyl chlorophosphate with different nucleophiles including water, ethanol, phenol and diethylamine were 0.35·10^−4^, 0.12·10^−5^, 0.38·10^−3^ and 0.28·10^−1^ L mol^−1^s^−1^, respectively. These data do not take into account the effect of an organic base present in the reaction media of an AT reaction. It reveals, however, that the secondary amine is much more reactive than phenol, water and ethanol in a reaction with chlorophosphate. Consequently, the existence of the trichloromethylphosphonate as an intermediate species in the experiments of Feringa et al. must be considered with caution because several experimental details are in favor of the existence of a competitive process that could, actually, be in favor of the addition of the nucleophilic amine on chlorophosphorinane as intermediate.

**Scheme 7:**
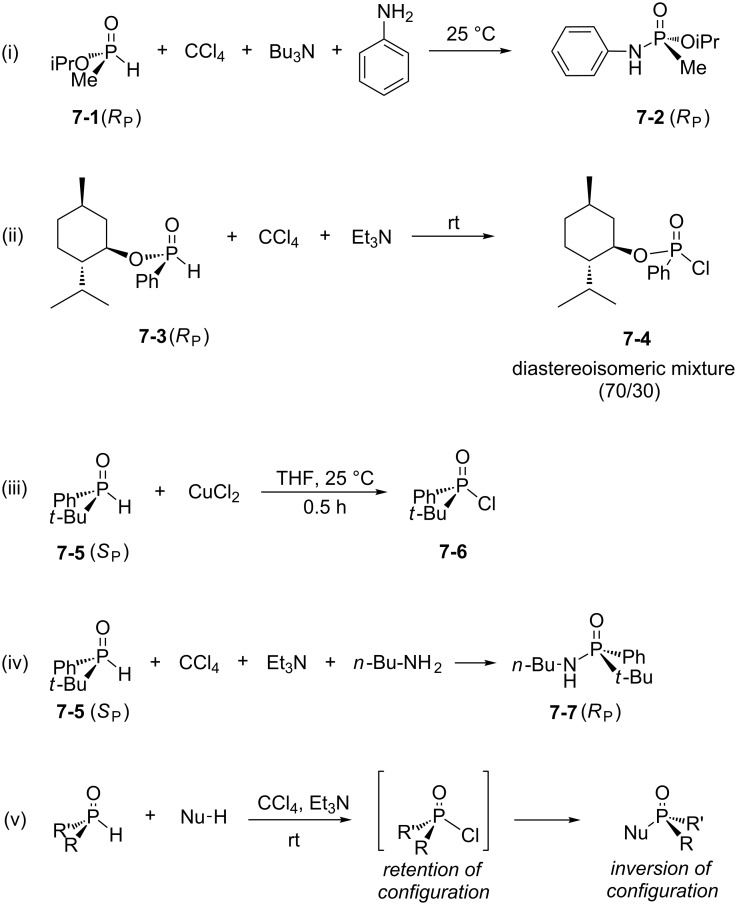
Selection of results that address the question of the stereochemistry of the AT reaction (adapted from [29–32]).

All the studies reported above shed some light on the mechanism of the AT reaction. None of these studies, however, focused on the chirality at the phosphorus atom. The first investigation of the stereochemistry of the AT reaction was reported by Reiff and Aaron [29]. They used enantiopure *O*-isopropyl methylphosphonite **7-1** (Scheme 7-i) as a substrate. This substrate was purified by distillation with no racemization. However, rapid racemization was observed when **7-1** was treated with sodium methoxide in methanol. The use of **7-1** in the AT reaction in the presence of CCl_4_, tributylamine and aniline as a nucleophile produced **7-2** with full stereocontrol (note: due to the substitution of a hydrogen atom by an aniline moiety, the numbering used to determine the chirality at the phosphorus atom was changed; consequently, the (*R*) configuration is preserved in the course of this reaction). The authors also observed a racemization (Scheme 7-ii) when the same reaction was achieved without the addition of a nucleophile (aniline in the present case). The use of similar reaction conditions (CCl_4_, NEt_3_) led Mikolajczyk et al. [30] to observe the formation of a mixture of diastereoisomers due to a racemization at the phosphorus atom. However, this racemization was not always observed which points out the different stability of diastereoisomeric species (cyclic phosphite in the present case). Other conditions of chlorination such as the use of *N*-chlorosuccinimide or, as reported more recently, CuCl_2_ produced, in the latter case, **7-6** with full stereocontrol (Scheme 7-iii) as unambiguously determined by X-ray diffraction analysis [31]. The addition of nucleophilic species (amine, alcohol, alkyllithium, etc.) on **7-6** or other chlorophosphonites was stereocontrolled with the inversion of the configuration at the phosphorus atom. In another publication it is reported on the AT reaction starting from enantiopure (*S*)-phenyl *tert*-butyl phosphinous acid **7-5** (Scheme 7 iv) [32]. In that case the final product **7-7** was isolated with full stereocontrol. This result indicates that both steps (i.e., the chlorination and subsequently the substitution of the chlorine atom with the nucleophile) were stereocontrolled. The detailed mechanism of each step, which may involve penta-coordinated phosphorus intermediates [28], is not well established. From a stereochemical point of view, these results can be summarized as shown in Scheme 7-v.

Other recent studies have reported stereocontrolled AT reactions. Han et al. [33] described the preparation of optically active organophosphorus acid derivatives from menthyl-based H-phosphinates (or secondary phosphine oxides) and a nucleophilic species (amines or alcohols) under AT reaction conditions. The reaction proceeded with a full stereoinversion at the phosphorus atom and led to the isolation of optically pure P–N coupling products with nearly quantitative yields (94%). The chemical structure and the stereochemistry of the products were unambiguously established by X-ray diffraction analysis. It is noteworthy that such coupling conditions have been extended to a wide variety of substrates including nitro-, methoxy-, trifluoromethylphenol or thiophenol with an almost similar reactivity. However, an exception was made when considering the reaction with aliphatic thiols (e.g., *n*-alkylthiols) [33]. In all studied cases, the authors concluded that the first step (i.e., the formation of a phosphoryl chloride intermediate) was achieved with retention of configuration (Scheme 7-v). Then, a subsequent attack of the nucleophilic substrate (amine, alcohol or thiophenol) occurred at the opposite side of the phosphorus–chlorine bond to afford the substitution product with high stereospecificity and an inversion of configuration.

**Scheme 8:**
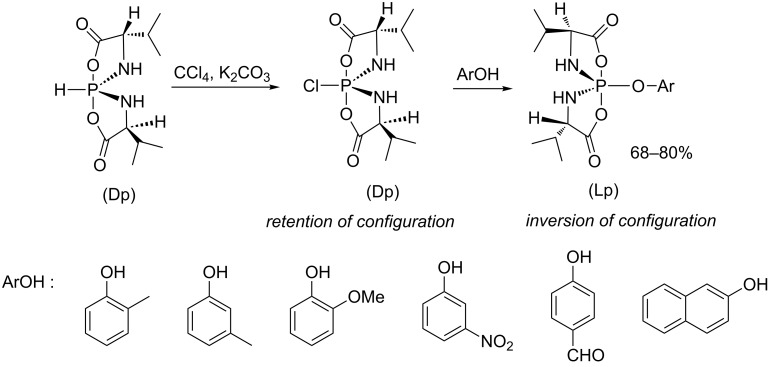
Synthesis of phenoxy spirophosphorane by the AT reaction (adapted from [34]).

Zhao et al. have also investigated the stereochemistry of the AT reaction by using chiral valine hydrospirophosphorane as a substrate and phenol derivatives as nucleophiles [34]. The mechanism described in their publication suggested the formation of a chlorinated spirophosphorane species as an intermediate, which retains the configuration at the phosphorus center. This hypothesis is strongly supported by X-ray diffraction analysis of a single crystal structure of the P–Cl intermediate species (Scheme 8). Thereafter, the reaction with phenol proceeds with an inversion of the configuration after a nucleophilic attack at the opposite side of the phosphorus–chlorine bond.

**Scheme 9:**
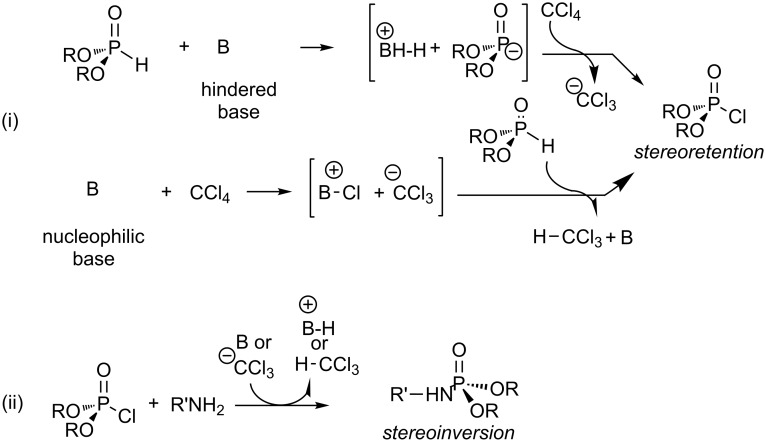
Suggested mechanism of the Atherton–Todd reaction, (i) and (ii) formation of chlorophosphate with a hindered or nucleophilic base; (iii) nucleophilic substitution at the phosphorus center with stereoinversion.

In conclusion, all studies which have investigated the mechanism of the Atherton–Todd reaction resulted in a summary of the likely reaction mechanism in Scheme 9. This mechanism seems to be general, but a different mechanism can take place when dimethyl phosphite is used due to its reaction with amine as shown in Scheme 4. On the basis of all these results, the hypothesis of the formation of dialkyl trichloromethylphosphonate as an intermediate of the Atherton–Todd reaction must be discarded.

### 3. Scope and synthetic conditions

**Scheme 10:**
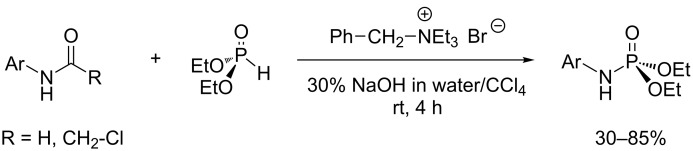
AT reaction in biphasic conditions (adapted from [38]).

CCl_4_ was the first solvent used in AT reactions. It plays a double role since it also acts as a halogenating agent that reacts with phosphite to produce chlorophosphate as an intermediate. However, many other solvents, including dichloromethane, chloroform, diethyl ether [35], THF [31], acetonitrile [34], DMF [36] and toluene [37] were also used. In that case a stoichiometric amount of the halogenating agent (CCl_4_, CBrCl_3_) is usually employed. Among these solvents, those that are not miscible with water are usually a good choice (e.g., CH_2_Cl_2_, CHCl_3_). At several occasions, alcohol (methanol, ethanol) was also used as a solvent even though it may compete with the nucleophilic species. This choice was made when the reagents were poorly soluble in other solvents (e.g., reagents like ammonium chloride salts). When this type of solvent is employed, the addition order of reagents can influence the result. Indeed, it is better to add CCl_4_ (or CBrCl_3_) and the tertiary amine at the end to be sure that the nucleophile (e.g., an amine) will be present when the chloro- or bromophosphate derivatives will be formed in situ. The mixture of reagents is usually achieved at 0–5 °C. Then, an additional stirring period of a few minutes to a few hours at rt (20 °C) is applied. The use of an anhydrous solvent is usually preferred in order to avoid the hydrolysis of chlorophosphate to pyrophosphate or phosphate. It is also possible to add molecular sieves (4 Å) to the reaction medium to remove traces of water, which can be useful when a poor nucleophilic species is employed. However, there are also a few reports on AT reactions in water–organic solvent mixtures when reactive nucleophilic species were used (primary amine) and under biphasic conditions with a phase-transfer agent as shown in Scheme 10 [38]. In this study, water and CCl_4_ were used to produce the biphasic system. NaOH is the base and benzyltriethylammonium bromide acted as a phase-transfer agent. This procedure was applied to the synthesis of *N*-arylphosphoramidate. It was observed that the classical AT reaction (CCl_4_, base, anhydrous solvent) applied to ortho-substituted anilines was not successful. However, the use of formanilide (R = H) or chloroacetanilide (R = CH_2_Cl) as a substrate (Scheme 10) produced the expected phosphoramidate by a mechanism which simultaneously involved deacetylation. The higher reactivity of formanilide and chloroacetanilide, when compared to aniline, was explained by the higher acidity of the remaining N–H bond. But the isolated yields were usually modest (50–70%) and only occasionally high (85%). With respect to the reactivity of chlorophosphate with water, Strawinski et al [21] reported that chlorophosphate can be placed in THF with 0.5 equiv of water without any trace of degradation but the addition of a base immediately gives rise to the formation of pyrophosphate (^31^P NMR around −12 ppm alkyl pyrophosphate and −25 ppm tetraaryl pyrophosphate) and to phosphate in the presence of additional water (^31^P NMR dialkyl phosphate 0.4 ppm and diaryl phosphate −9.6 ppm). With the use of a biphasic system it is likely that the reaction takes place in the CCl_4_ phase because all the reagents, except the hydroxide anion, are soluble in CCl_4_. The hydroxide anion is actually transferred to the organic phase thanks to the phase-transfer agent.

**Scheme 11:**
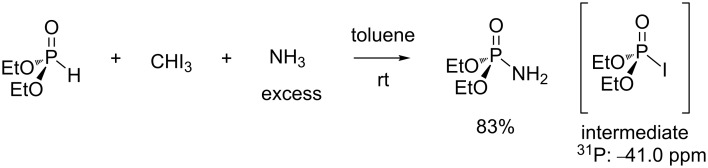
AT reaction with iodoform as halide source (adapted from [37]).

Different halogenating agents were used to produce in situ chlorophosphate from phosphite. CCl_4_ was used systematically for a long period of time. However, Atherton and Todd [2] have shown that reaction rates were increased by using CBrCl_3_. This last halogenating agent is likely the reagent of choice when a stoichiometric amount or a slight excess is employed. Other halogenating agents were studied for the AT reaction. CBr_4_ [39] is also able to produce reactive bromophosphate as an intermediate species. However, it is more expensive compared to CBrCl_3_ and, to the best of our knowledge, better results with the use of CBr_4_ have not been published yet. The AT reaction was also reported with the use of trichloroacetonitrile as a halide source [35]. Accordingly, the chlorophosphate was formed in the presence of triethylamine. Iodoform (CHI_3_) is another reagent that can be used as a halide source for the AT reaction [37]. As shown in Scheme 11, gaseous anhydrous ammonia was passed through a solution of diethyl phosphite and iodoform in toluene to produce the expected phosphoramidate in good yield (83%). In an independent experiment the authors have identified the iodophosphate (^31^P at −41.0 ppm), a compound which decomposes when stored for a few hours at rt.

**Scheme 12:**
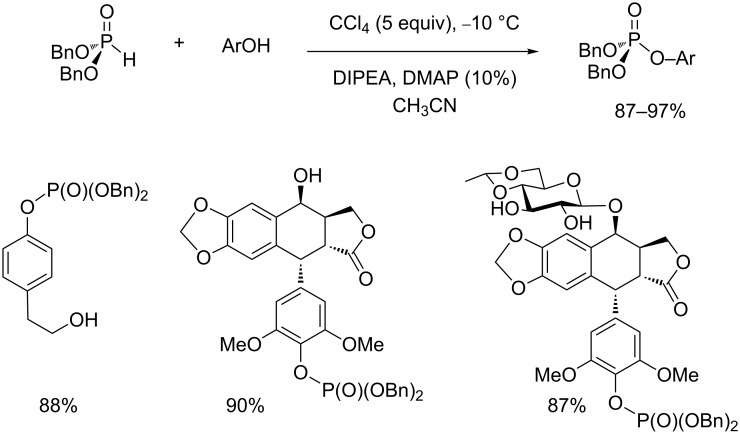
AT reaction with phenol at low temperature in the presence of DMAP (adapted from [40]).

With respect to the base used for the AT reaction, it can be noticed that a trialkylamine is generally the best choice. This amine is frequently triethylamine or diisopropylethylamine (Hünig’s base, DIPEA). It must be noted that the use of this hindered base (DIPEA) could favor the mechanism detailed in Scheme 3 instead of a mechanism that would involve a nucleophilic attack of the amine on the alkyl chain of the phosphite (Scheme 4). Some authors add catalytic quantities of dimethylaminopyridine (DMAP). For instance, this procedure was used to produce arylphosphate by reacting phenol with dibenzyl phosphite [40]. The presence of DMAP and limited excess of CCl_4_ (5 equiv) produced the expected aryl phosphate in excellent yield even at a low temperature (−10 °C). Interestingly, this study illustrates that the AT reaction is chemoselective since only the phenol reacts even in the presence of primary or secondary alcohols (Scheme 12).

**Scheme 13:**
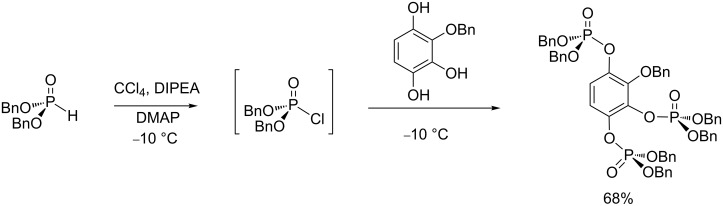
Synthesis of a triphosphate by the AT reaction starting with the preparation of chlorophosphate (adapted from [41]).

With the AT reaction, the order of addition of reagents can impact its efficacy. Usually, the phosphite or the halide source (CBrCl_3_) is added dropwise to a mixture of the nucleophile (amine, phenol), trialkylamine (e.g., DIPEA) and a solvent cooled at −10 °C to 5 °C. According to this procedure, the chlorophosphate formed as an intermediate immediately reacts with the nucleophile already present in the reaction medium. It is noteworthy that another synthetic procedure consists to firstly prepare the chlorophosphate and then add the nucleophilic species (Scheme 13) [41]. Following this procedure, chlorophosphate was first prepared at a low temperature (−10 °C) by reacting dibenzyl phosphite with CCl_4_ in the presence of DIPEA and a catalytic amount of DMAP. Then, the nucleophile (in that case a phenol) is added dropwise to the chlorophosphate solution at −10 °C to produce, after purification, the triphosphate in 68% yield. Generally, the first protocol is preferred to avoid any hydrolysis of chlorophosphate, which could be explained by the presence of trace amounts of water.

As indicated above, a tertiary amine is the preferred base for the AT reaction. However, primary or secondary amines can be also used when they simultaneously act as a nucleophile and a base. In that case, two equivalents of amine must be added (Scheme 11). The use of NaOH as a base was also reported (exemplified in Scheme 10) in a protocol involving phase-transfer catalysis.

**Scheme 14:**
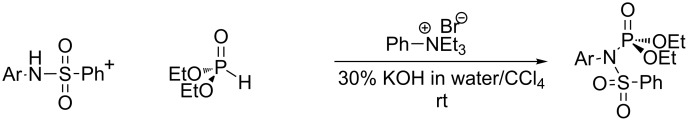
AT reaction with sulfonamide (adapted from [42]).

Initially, the nucleophile engaged in the AT reaction was a primary or secondary alkylamine. However, the use of other nucleophilic species was reported. First, as illustrated above, aniline can be used. However, ortho-substituted aniline reacts with more difficulties according to Lukanov et al. [38]. Indeed the aniline must be activated as a sulfonamide (or acetamide) to produce the expected phosphoramidate [42]. The authors, who used a phase-transfer agent, postulated that this activation resulted from the increase of the acidity of the N–H bond despite the evident reduction of the nucleophilic character (Scheme 14).

**Scheme 15:**
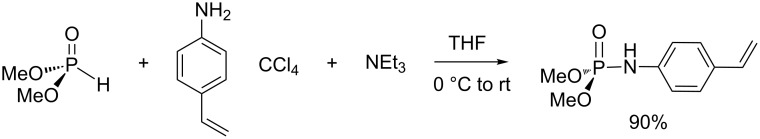
Synthesis of a styrylphosphoramidate starting from the corresponding aniline (adapted from [43]).

To further illustrate AT reactions with aniline, the work of Dumitrascu et al. [43] is worth mentioning. Recently, they have reported on the preparation of an aryl phosphoramidate with a styryl moiety from the corresponding aniline (Scheme 15). The phosphoramidate was isolated with an excellent yield (90%), and this product was subsequently used to prepare polymers.

**Scheme 16:**
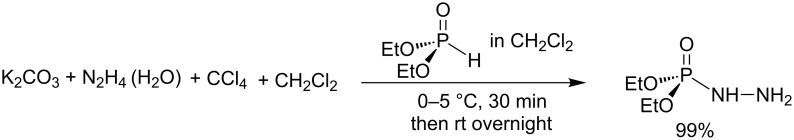
Use of hydrazine as nucleophile in AT reactions (adapted from [48]).

Other nitrogen-based nucleophiles were also engaged in AT reactions. Hydrazine is a substrate which produces phosphoramidate in high yield. The first example, reported by Prokof’eva et al. [44], used arylhydrazine as a substrate. The reaction proceeded in CCl_4_ in the presence of triethylamine and produced phosphoramidates in 60–82% yield. Unsubstituted hydrazine can also be used as a nucleophile in the AT reaction [45–47]. The best synthetic conditions employed phase-transfer catalysis [48]. Accordingly, CCl_4_ was used as a solvent or co-solvent, triethylbenzylammonium chloride acts as a phase-transfer agent, and K_2_CO_3_ was used as a base. Hydrazine hydrate was the source of hydrazine. The expected diethoxyphosphinylhydrazine was isolated in almost quantitative yield (99%) after a purification step by liquid/liquid extraction (Scheme 16). This procedure was also recently used by Matthews et al. to produce the same compound (diethoxyphosphinylhydrazine) in a slightly lower yield (90%) [49]. *O*-Methoxyhydroxylamine is another nitrogen-based nucleophile engaged in AT reactions. The first example reported by Wadsworth et al. produced low yield [50]. The procedure was improved by the addition of a phase-transfer agent (triethylbenzylammonium chloride). Accordingly, a series of phosphorylated *O*-alkylhydroxylamine was produced in medium to good yield (45–97%) [51].

**Scheme 17:**
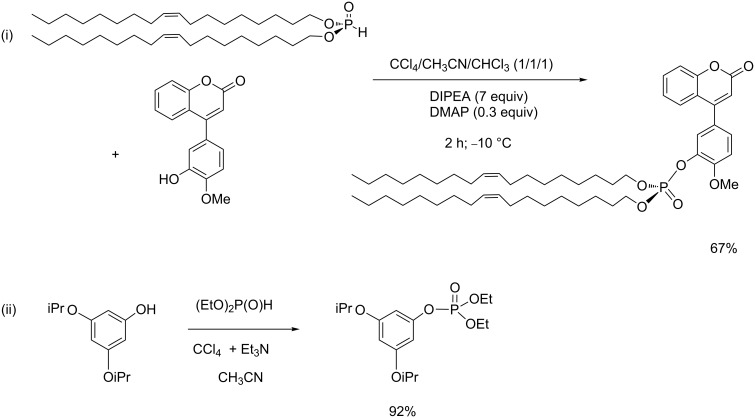
AT reaction with phenol as a nucleophilic species; synthesis of dioleyl phosphate-substituted coumarine derivative (i) and synthesis of diethyl aryl phosphate (ii) (adapted from [55] and [56]).

The use of alcohol as a nucleophile has been rarely reported indicating that its nucleophilicity is not good enough to produce the expected phosphate in good yield. This reaction was observed when CBrCl_3_ or CBr_4_ were used as a halide source according to the initial works of Atherton and Todd [1–2]. Nevertheless, this reaction is limited and successful only with an alcohol such as methanol or ethanol. In our group we have occasionally employed methanol as a solvent for selected reactions due to the low solubility of certain substrates (e.g., amino acid hydrochlorides). In that case, a noticeable amount of diethyl methyl phosphate was observed originating from the reaction of methanol on the chlorophosphate intermediate. Because the AT reaction is not efficient to produce alkylphosphate from alkylalcohol, an alternative is the usage of chlorophosphate in the presence of a Lewis acid catalyst. For this purpose, Ti(*t*-BuO)_4_ was identified as an effective catalyst [52–53]. The use of phenol as a nucleophilic species resulted in better yields. Hence, the AT reaction applied to a substrate possessing both alcohol and phenol functional groups, produces the phosphate that engaged the phenol function as shown in Scheme 12. AT reactions with phenol are further illustrated by the work of Charette et al. who used this reaction in the first step of a multistep synthesis to produce chiral arylphospholane [54]. Another interesting example, reported by Selikhov et al., involved an AT reaction between a functionalized coumarine and dioleyl phosphite [55]. In this reaction the authors used a mixture of a solvent (CH_3_CN/CHCl_3_) and an excess of CCl_4_. DIPEA and a catalytic amount of DMAP were also added in the reaction medium. The expected phosphate was isolated with 67% yield (Scheme 17-i). Better yields are usually obtained with phosphite possessing two shorter alkyl chains as exemplified by a reaction reported by Taylor et al (Scheme 17-ii) [56].

**Scheme 18:**
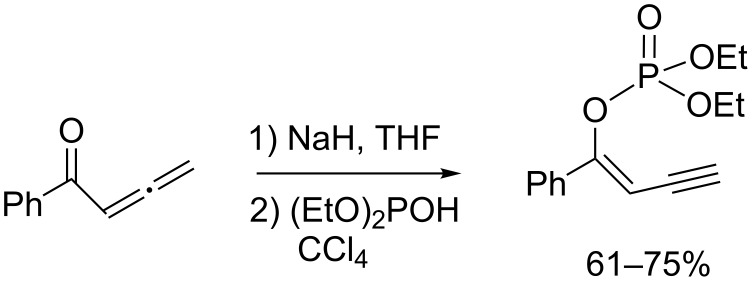
Synthesis of β-alkynyl-enolphosphate from allenylketone with AT reaction (adapted from [58]).

The phosphorylation of phenol by the AT reaction was also reported under biphasic conditions in the presence of a phase-transfer agent. Interestingly, the study of Ilia et al. [57], reports the use of both liquid/liquid and liquid/solid biphasic systems. They observed that the second reaction conditions (i.e., the ones without the addition of water) produced better results with yields between 72 and 86%. In this reaction NaOH or K_2_CO_3_ was used as a base, tetrabutylammonium bromide as a phase-transfer agent and CCl_4_ as a reagent and solvent. Finally, enolate is another nucleophilic species that was engaged in the AT reaction as shown in Scheme 18. In this reaction, an allenylketone was mixed with NaH in THF at −10 °C. After deprotonation (30 min), diethyl phosphite in CCl_4_ was added dropwise. Treatment with acetic acid and purification on silica gel finally afforded the β-alkynyl-enolphosphate in 61–75% yield [58].

**Scheme 19:**
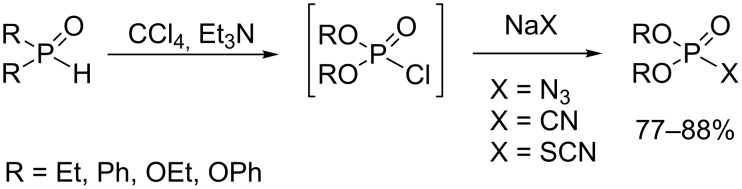
Synthesis of pseudohalide phosphate by using AT reaction (adapted from [67]).

Azide, nitrile and thiocyanate were three other nucleophilic species used to produce pseudohalogenated phosphorus species by the AT reaction. Among them, the commercially available diphenyl phosphorazidate (DPPA) and diethyl phosphorocyanidate (DEPC) are widely employed as peptide coupling reagents [59–62]. DPPA was also used in many organic reactions such as the Curtius rearrangement [59], the thiol ester synthesis [63], the azidation of alcohol and phenol [64], and the synthesis of phosphoramidate [65]. The most common way to prepare pseudo-halogenated phosphorus species involves a nucleophilic substitution on chlorophosphate with sodium salts (NaN_3_, NaCN or NaSCN) [66]. Due to the reactivity of chlorophosphate and its difficulty of storage, its production in situ by the AT reaction was studied by Shi et al. as depicted in Scheme 19 [67]. (RO)_2_P(O)Cl is generated from phosphite (RO)_2_P(O)H, CCl_4_ and a catalytic amount of Et_3_N. This chlorophosphate is immediately engaged in a reaction with a sodium salt NaX (X = N_3_, CN or NCS) to afford the pseudo-halide phosphorus species in satisfactory yields (77–88%). Interestingly, the same authors reported that pseudo-halide phosphate can also be obtained by using phase-transfer catalysis [68]. Accordingly, NaX (X = N_3_, CN or NCS) reacts with dialkyl phosphate in water–organic solvent mixtures under phase-transfer catalysis. The catalyst was either TBAB (tetrabutylammonium bromide), TBAI (tetrabutylammonium iodide), TMAC (tetramethylammonium chloride) or 18-crown-6. The best result was obtained with TBAB and 18-crown-6 catalysts in a CH_2_Cl_2_/water or AcOEt/water mixture (yields included from 72 to 85%).

**Scheme 20:**
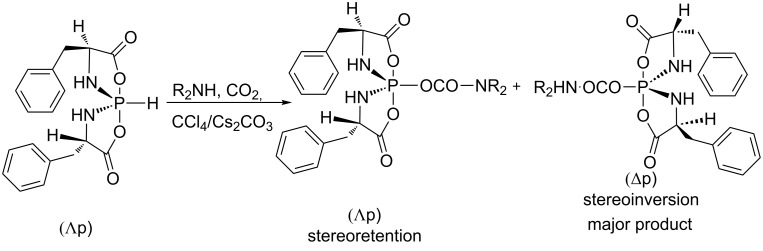
AT reaction with hydrospirophosphorane with insertion of CO_2_ in the product (adapted from [69]).

The carbamate anion is another nucleophilic species recently engaged in the AT reaction with hydrospirophosphorane as substrate [69]. This nucleophilic species was generated in situ by reacting a secondary amine and CO_2_. Then, it reacted with chlorospirophosphorane produced in situ from hydrospirophosphorane, CCl_4_ and Cs_2_CO_3_ (Scheme 20). This reaction can be viewed as an activation of CO_2_ by an amine to produce the nucleophilic carbamoyl moiety. The major product of this reaction corresponds to an inversion of the configuration at the phosphorus with diastereoisomeric excess included from 6.8 to 1. It is noteworthy that the usage of a primary amine as an educt in this reaction did not lead to the insertion of CO_2_ , and the product resulted from the reaction of the amine on the chlorospirophosphorane. This result may be explained by the higher nucleophilicity of the primary amine in this reaction media.

**Scheme 21:**
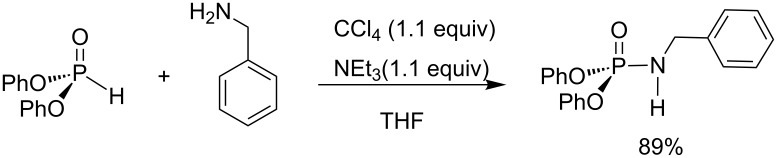
AT reaction with diaryl phosphite (adapted from [70]).

The phosphorus species engaged in AT reactions mainly include dialkyl phosphites. It must be noted that the alkyl-chain length can be changed and extended to lipid chains as exemplified in Scheme 17 (oleyl chains). Diaryl phosphite can also be used for AT reactions as illustrated in a recent example published by Gaan et al. (Scheme 21) [70].

**Scheme 22:**
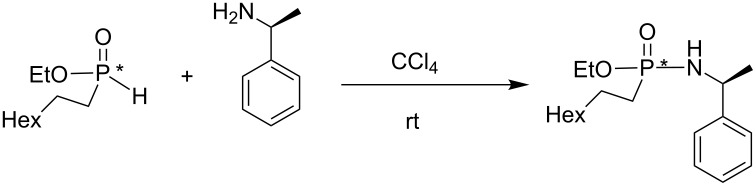
AT reaction with O-alkyl phosphonite (adapted from [71]).

*O*-Alkyl phosphonite can also be engaged in AT reactions as recently illustrated by Montchamp et al. in a study dedicated to the hydrophosphinylation of terminal alkenes (Scheme 22) [71]. Shi et al. have also reported the reactivity of *O*-alkyl arylphosphonite in AT reactions with azide as the nucleophilic species [67].

**Scheme 23:**
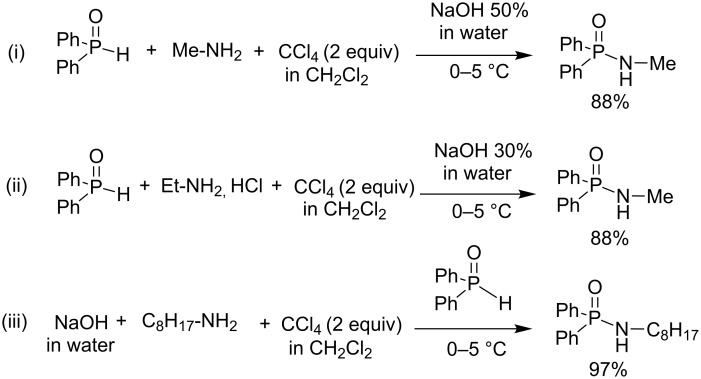
Use of phosphinous acid in AT reactions (adapted from [72]).

Phosphinous acid (R_2_POH) was less extensively studied as a phosphorus-based substrate in AT reactions. However, one full study was reported by Bondarenko et al. [72]. These authors reported three different methods to produce phosphinic amides by reacting primary or secondary amines with phosphinous acid in the presence of CCl_4_ and aqueous NaOH. The first protocol was applied when methylamine or ammonia was used as a nucleophile (Scheme 23-i). In that case, a 50% aqueous solution of NaOH was slowly added to a biphasic solution composed of aqueous methylamine, CCl_4_ and dichloromethane. The second protocol (Scheme 23-ii) is almost similar to the first one except that a hydrochloride salt (e.g., ethylamine hydrochloride) was used as a substrate without prior solubilization in water. Finally, for the third protocol (Scheme 23-iii), the phosphinous acid is added to a mixture of the other substrates placed in a biphasic solution (water/CH_2_Cl_2_). The yields were between 77 to 97%.

**Scheme 24:**
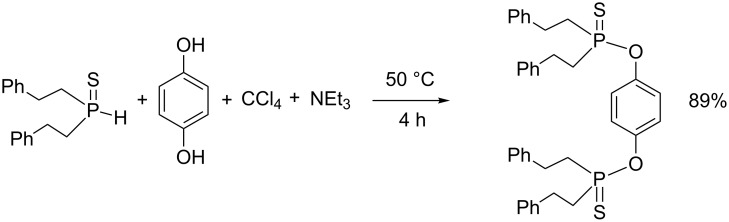
AT reaction with secondary phosphinethiooxide (adapted from [76]).

Thiophosphorus-based precursors can also be engaged in AT reactions. Recently, secondary phosphinethiooxide has been engaged as a phosphorus-based precursor in the AT reaction [73]. The reaction proceeds in good yield (80%) with CCl_4_ as a solvent, an amine as a nucleophile and triethylamine as a base. The use of secondary phosphineselenoxide was also reported by the same authors but the yields were lower (35–38%) [74]. The procedure was also applied to alcohol [75] or diphenol derivatives acting as a nucleophile as shown for hydroquinone in Scheme 24 [76]. *O,O*-dialkyl thiophosphite was also engaged in AT reactions to produce dinucleotide designed as an anti-HIV prodrug [77].

**Scheme 25:**
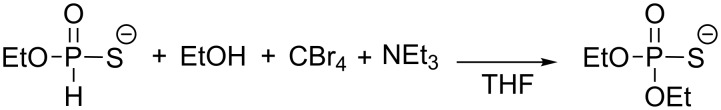
Use of H-phosphonothioate in the AT reaction (adapted from [78]).

H-phosphonothioates are intermediates considered for the synthesis of nucleotide analogues. This functional group can be used as a nucleophilic species in a coupling reaction with a primary alcohol. This coupling reaction is usually achieved by I_2_ acting as an oxidant, but Stawinski et al. also tested other halide sources including CCl_4_ and CBr_4_ [78]. Despite iodine is the reagent of choice in this reaction, CBr_4_ is also efficient to produce the coupling product (Scheme 25), whereas CCl_4_ was inefficient.

AT reactions usually proceed at 0–5 °C but lower temperatures (−10 °C [41]) and higher temperatures (50°C [76]) were also reported. The use of microwave (MW) activation was also reported by Beletskaya et al. [79]. This activation was applied to AT reactions which used α-aminophosphonate as a nucleophile in the presence of CCl_4_ and triethylamine. The authors reported that no reaction occured at rt, whereas phosphoramidate was obtained in low yield (15–20%) when classical heating (24 h, 110 °C) was applied. However, phosphoramidates were isolated in 63–93% yields with MW heating. The reaction time was significantly shorter with MW since a full conversion was reached after only 30 to 40 minutes of heating.

**Scheme 26:**
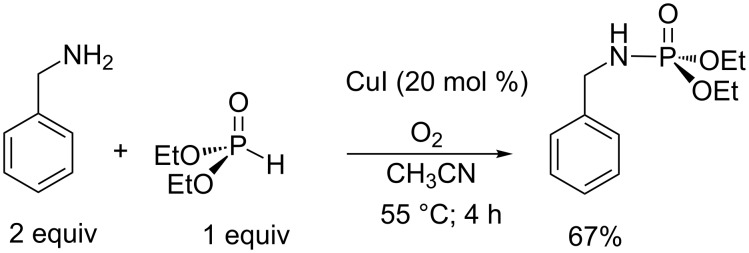
AT-like reaction with CuI as catalyst and without halide source (adapted from [80]).

The recent works of Hayes et al. reported on the synthesis of phosphoramidates starting from dialkyl phosphite and primary amine without a halogen source [80]. Instead of using CCl_4_ or CBr_4_, the authors employed a catalytic amount of CuI as depicted in Scheme 26. These new synthetic conditions, that required O_2_ for the reoxidation of copper, can be viewed as an extension of the AT reaction which facilitates the avoidance of a halide source. Even though the yield is usually lower compared to classical AT conditions, this result opens new perspectives for the development of green processes.

### 4. Applications

#### 4.1 Synthetic utilities of AT reactions

In this section we will discuss the use of AT reactions to produce aryl phosphates or phosphoramidates which are important intermediates for a variety of subsequent transformations or can be used as organocatalysts.

**Scheme 27:**
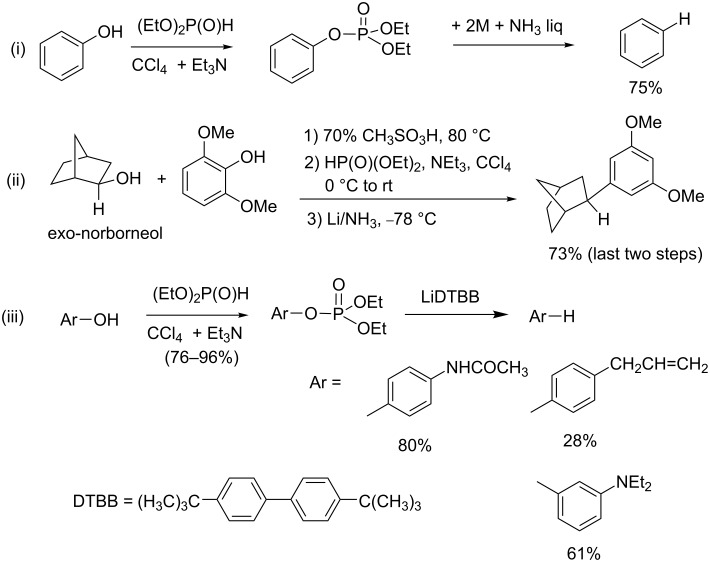
Reduction of phenols after activation as phosphate derivatives (adapted from [81] i ; [82], ii; and [83], iii).

Aryl dialkyl phosphates, prepared by the AT reaction from phenol derivatives, were employed to reduce phenol functional groups. It is noteworthy that phenol compounds and their related deoxygenated derivatives represent an important family of natural or synthetic compounds exhibiting, for example, antitumor or antiallergic activities. The reduction of a phenol group implies its activation as a good leaving group. Phosphate is one of the possible activating groups. Its use for the desoxygenation of phenol was first reported by Kenner et al. [81] who implemented the reaction with liquid ammonia as a solvent in the presence of metal as a reducing agent (e.g., Na) (Scheme 27-i). A similar methodology was used to produce 2-substituted bornane in a three-step sequence as shown in Scheme 27-ii [82]. Firstly, a Friedel–Crafts reaction produced the para-substituted phenol in 42% yield. Then, the phenol group was transformed in diethyl aryl phosphate with an AT reaction in 99% yield. Finally, the reduction of the phosphate was achieved in 74% yield. Similar synthetic schemes (AT reaction and reduction with Li/NH_3_) were also reported in another publication [83]. This procedure was recently improved by Lusch et al. (Scheme 27-iii) by using a lithium di-*tert*-butylbiphenylide radical anion [84]. Accordingly, a wide panel of phenol derivatives was first transformed in diethyl aryl phosphate with the AT reaction (yields from 76 to 96%), and then these phosphates were reduced to the corresponding aromatic hydrocarbons with moderate to good yields (10 to 83%).

**Scheme 28:**
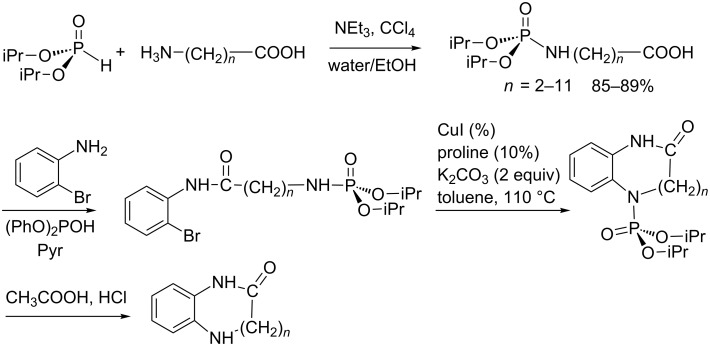
Synthesis of medium and large-sized nitrogen-containing heterocycles (adapted from [85]).

The phosphoramidate group can also be used to enhance the reactivity of an amine. This activation role of phosphoramidates was illustrated for the synthesis of medium and large-sized cyclic diaza-compounds by Zhao et al. [85]. This sequence started with the AT reaction (Scheme 28) to produce ω-difunctionalized phosphoramidatecarboxylic acid. In the last step, the phosphoramidate group activates the amine which is cyclized by a copper-catalyzed N-arylation. In the last step, the phosphoramidate is cleaved by an acidic treatment. This sequence illustrates that the AT reaction is compatible with the presence of free carboxylic acid functional group.

**Scheme 29:**
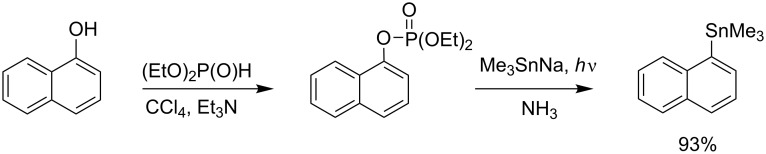
Synthesis of arylstannane from aryl phosphate prepared by an AT reaction (adapted from [86]).

Aryl dialkyl phosphate, easily prepared starting from phenol in an AT reaction, can also be transformed into arylstannane derivatives by a S_RN_1 reaction by the photostimulation of trialkyl [86] or triarylstannyl [87] ion in liquid ammonia as shown in Scheme 29. This example shows that the AT reaction was achieved with CCl_4_ as a halogenating agent. Then, the stannylation reaction produced the arylstannane in 75 to 90% yield [88].

**Scheme 30:**
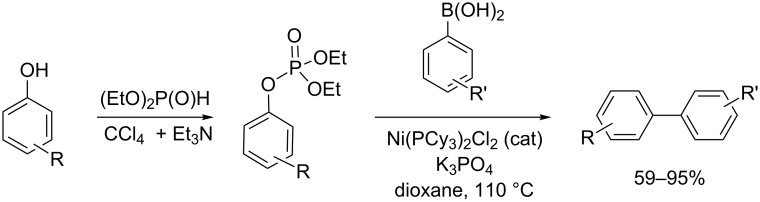
Synthesis and use of aryl dialkyl phosphate for the synthesis of biaryl derivatives (adapted from [89]).

The Suzuki–Miyaura cross-coupling reaction is another type of reaction with the phosphate group acting as a leaving group. As exemplified in Scheme 30, the aryl dialkyl phosphate was produced in good yield by an AT reaction [89]. Then, this phosphate group reacted with aryl Grignard, as a Kumada–Tamao-Corriu cross-coupling, in the presence of a nickel catalyst [90].

**Scheme 31:**
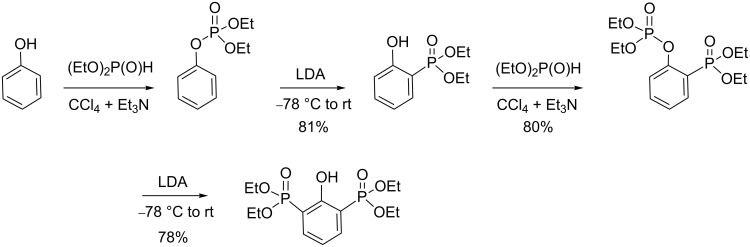
Synthesis of aryl dialkyl phosphate by an AT reaction from phenol and subsequent rearrangement yielding arylphosphonate (adapted from [91]).

Aryl dialkyl phosphate, which was readily obtained by the AT reaction from phenol as shown in Scheme 31 [91], can be employed for the production of aryl phosphonate by applying a phospho-Fries rearrangement (a rearrangement initially reported by Melvin et al. [92]). This rearrangement proceeds by an ortho-metallation that can be characterized at a low temperature [93]. Then, the *o*-lithium species rearranges to produce 2-hydroxyarylphosphonate [94].

The recent publication of Yang et al. [95] reports the amination of either triaryl phosphate or dialkyl aryl phosphate catalyzed by nickel organometallic complexes. It is an additional illustration of the use of aryl phosphate, which can be readily obtained by AT reactions.

**Scheme 32:**
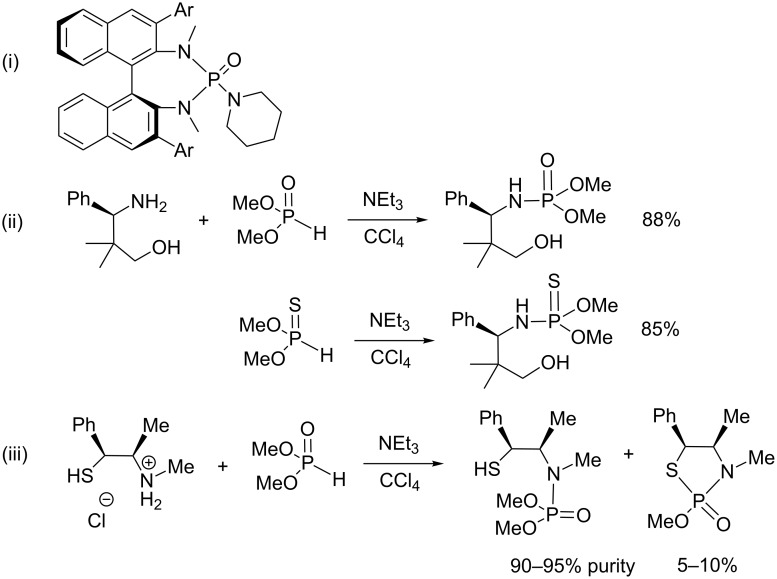
Selected chiral phosphoramidates used as organocatalyst; i) chiral phosphoramidate used in the pioneer works of Denmark et al. [101]; ii, iii) synthesis of organocatalysts by using AT reaction (adapted from [107]).

Beside the use of phosphorus species as a substrate in reactions as those reported above, phosphoramidates, prepared by AT reactions, can also act as an organocatalyst. Indeed, with the strongly polarized P–O bond on one hand, and the P–N or P–NH bond on the other hand phosphoramides are good Lewis bases [96–98]. Hexamethylphosphoric triamide (HMPA) (or analogues) was the first phosphoramide derivative that was extensively studied as an organocatalyst [98–99]. However, HMPA was classified as a human carcinogen [100]. One of the first examples of the use of chiral phosphoramide ligands (Scheme 32-i) in organocatalysis was described by Denmark et al. who studied the enantioselective crossed aldol reaction of aldehydes with trichlorosilyl enol ethers. They obtained the aldol products in high yields with moderate to good enantioselectivities [101]. The chiral phosphoramidates used in this study and tested in many other enantioselective reactions (aldol reaction [102], Michael addition [103], Diels–Alder reaction [104], Friedel–Crafts alkylation [105]) illustrate the use of phosphoramidates as organocatalysts. These phosphoramidates were not synthesized by an AT reaction. Instead, the reaction of an amine with an electrophilic phosphorus species (P–Cl based compounds) afforded the phosphoramidates. Only a few examples (shown in Scheme 32-ii,iii) of chiral phosphoramidates were synthesized with the AT reaction likely due to the commercial accessibility of some chlorophosphine or chlorophosphate that render the AT reaction pathway less attractive. However, as reported in Scheme 32-ii, some chiral phosphoramidates and thiophosphoramidates can be readily synthesized with the AT reaction in high yield (85–88%) from dimethyl phosphite or *O*,*O*-dimethyl thiophosphite [106]. When the chiral amine was functionalized with a thiol group (Scheme 32-iii), the expected phosphoramidate was jointly isolated with 5 to 10% of thiophosphoramidate resulting from a cyclization reaction. The chiral phosphoramidates (Scheme 32-ii and iii) were tested as a chiral catalyst for the nucleophilic addition of diethylzinc [107] on benzaldehyde or for the asymmetric borane reduction of a prochiral ketone. The phosphoramidate (Scheme 32-iii) was the most efficient catalyst for these two reactions (ee: 95–98%, conversion 87–98%).

**Scheme 33:**
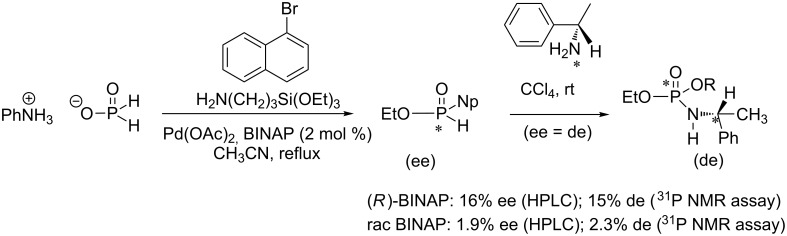
Determination of ee of H-phosphinate by the application of the AT reaction with a chiral amine (adapted from [108]).

In relation with asymmetric synthesis, the determination of the enantiomeric excess (ee) is usually achieved by different methodologies including chiral HPLC or CPG. In a recent study, Montchamp et al. used the AT reaction for the determination of the ee of P-chiral H-phosphinates by the formation of diastereoisomers (Scheme 33) [108]. The ee determined by this method, which can be achieved directly in the NMR tube before recording ^31^P NMR spectra, was consistent with those determined by other methods (e.g., chiral HPLC).

#### 4.2 Flame retardants

**Scheme 34:**
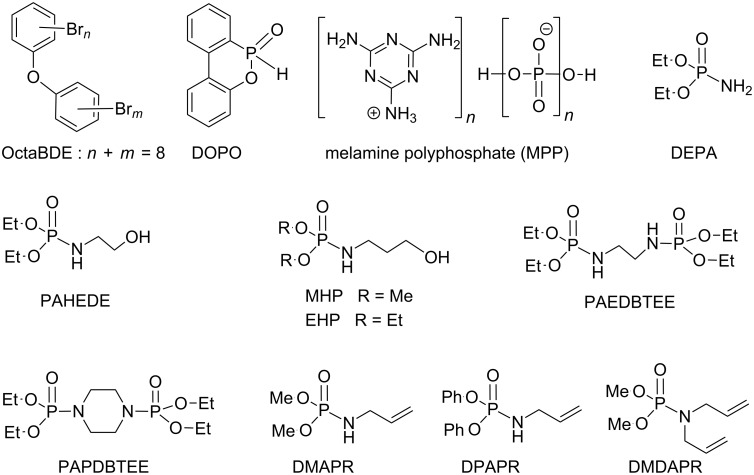
Chemical structure of selected flame retardants synthesized by AT reactions; (BDE: polybrominated diphenyl ether; DOPO: 9,10-dihydro-9-oxa-10-phosphaphenanthrene-10-oxide; DEPA: diethyl phosphoramidate; PAHEDE: phosphoramidic acid-*N*-(2-hydroxyethyl) diethyl ester; EHP: diethyl 3-hydroxypropylphosphoramidate; MHP: dimethyl 3-hydroxypropylphosphoramidate; PAEDBTEE: phosphoramidic acid-1,2-ethanediylbis-tetraethyl ester; PAPDBTEE: phosphoramidic acid-1,4-piperazinediyl tetraethyl ester; DMAPR: dimethyl allylphosphoramidate; DPAPR: diphenyl allylphosphoramidate; DMAPR: dimethyl diallylphosphoramidate).

Since the early age of the development of synthetic fibers, the production of flame retardants became an industrial and academic challenge aiming to identify efficient compounds for this purpose but also to elucidate the mechanisms involved in the burning process of polymers. This goal was also extended, for evident safety reasons, to natural fibres (e.g., cotton). Halogen-based flame retardants were extensively employed (e.g., OctaBDE) but for safety reasons that pointed out carcinogenic risks and/or to the production of toxic fumes when burning (e.g., HBr) several of these halogenated flame retardants were withdrawn from the market (e.g., octaBDE) [109]. Phosphorus-based compounds [110] are another class of flame retardants. This type of compounds contributes to the extinction of the flame by, at least, two distinct mechanisms. First, some phosphorus compounds (e.g., DOPO, Scheme 34) act on the gas phase by the production of non-flammable compounds (e.g., water) or by the production of reactive species that act as hydroxyl radical scavengers. Alternatively, phosphorus-based flame retardants may act on the solid phase by forming a thermal barrier between the solid phase and the gaseous phase. This charring process results from the formation of polyphosphoric acid derivatives. Interestingly, the association of phosphorus and nitrogen-based flame retardants proved to be efficient probably due to synergic effects. Melamine polyphosphate (MPP, Scheme 34), illustrates this possibility: melamine decomposes endothermically and produces NH_3_ when burning (a gas-phase active agent), while polyphosphate, which is simultaneously produced, favors the charring process. The synergic effects of phosphorus and nitrogen-based flame retardants led scientists to investigate the synthesis of organic compounds characterized by the presence of these two elements. Accordingly, phosphoramidates constitute an interesting family of potential flame retardants and the AT reaction is an efficient tool for the production of this type of compounds.

Phosphoramidate derivatives were investigated as flame retardants for many years [111–112]. However, some recent studies, which will be discussed below, have reinvestigated the use of this type of compounds. We focus on the synthetic procedures of phosphoramidates. These derivatives can be divided in two broad categories depending on the presence of polymerizable groups. When polymerizable groups are present, the fire-resistant molecule can be included in polymers by copolymerization or can act as a crosslinking agent. This type of compounds is also employed for the surface modification of fibers or polymers (e.g., plasma technique). Without a polymerizable group, phosphoramidates can be used as an additive in polymers. Gaan et al. [113] have studied the thermal decomposition of cotton cellulose treated with different phosphoramidates. More specifically, they have shown that a secondary phosphoramidate (e.g., PAHEDE) was more efficient than a primary phosphoramidate (DEPA; Scheme 34). Additional results reported by Rupper et al. [114] indicated that PAHEDE produced phosphoric acid moieties at the surface of cellulose after burning. Moreover, cellulose interacts with phosphoramides to produce C–O–P bonds. In these studies, secondary phosphoramidate (e.g., PAHEDE) was synthesized by reacting diethyl phosphite with ethanolamine in CCl_4_ and with one equivalent of triethylamine. After filtration and concentration, the phosphoramidate was isolated in quantitative yield after distillation. It is noteworthy that the synthesis is readily achieved and can be applied to large-scale productions. More recently, the comparison of the flame-retardant properties of MHP and EHP (Scheme 34), led to the conclusion that phosphoramidate with two methoxy groups (MHP) was more efficient [115]. The mechanism of degradation states that the MHP produced covalent bonds with cotton cellulose while the diethyl analogue (EHP) did not produce any such bonds. In this study, MHP was produced by an AT reaction as reported for PAHEDE [113]. For the synthesis of MHP, the aminoalcohol engaged in the AT reaction exhibited complete chemoselectivity since no trace amount of phosphate was reported. Some bis(phosphoramidates) were also considered as flame retardants. PAEDBTAEE and PAPBDTEE (Scheme 34) were compatible with cotton acetate and enhance the formation of char [116]. These two bis(phosphoramidates) were produced at a 0.1 mol scale (31 g of pure PAEDBTEE) with the AT reaction carried out in THF with a stoichiometric amount of CCl_4_ (0.2 mol) and diethylphosphite (0.2 mol). Yields greater than 95% were reported for these crystalline solids.

**Scheme 35:**
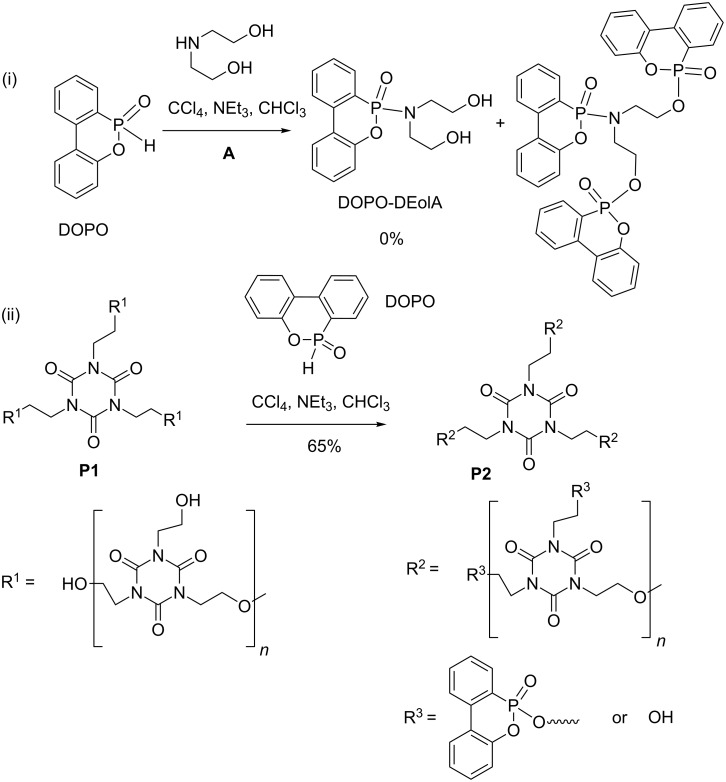
Transformation of DOPO (i) and synthesis of polyphosphonate (ii) by the AT reaction (adapted from [117] and [118]).

Other studies reported the modification of DOPO (a flame retardant in epoxy resins, Scheme 35-i) with the aim to react the P–H bond (phosphinate) to incorporate either nitrogen or oxygenated groups. Attempts to produce P–N bonds by using the AT reaction with diethanolamine (DEolA) failed because no chemoselectivity was observed between the secondary amine and the primary alcohol [117], whereas in other studies involving dialkyl phosphite a chemoselectivity was observed [107]. It can therefore be concluded that the phosphinate modifies the reactivity of the phosphorus group leading to this absence of selectivity. This surprising reactivity of DOPO with alcohol functional groups inspired the authors to produce cyclic phosphonates with the AT reaction. Accordingly, polymer P1, obtained by polycondensation was functionalized by an AT reaction to produce polymer P2 in 65% yield (Scheme 35-ii) [118].

All these recent studies, which report flame-retardant properties of phosphorus-based compounds illustrate that the AT reaction is easy to implement and, interestingly, high yields are usually obtained. Accordingly, phosphoramidates are cheap to manufacture and we assume that this reaction will be employed to design novel flame retardants.

#### 4.3. Phosphorus-based amphiphiles and biological applications

**Scheme 36:**
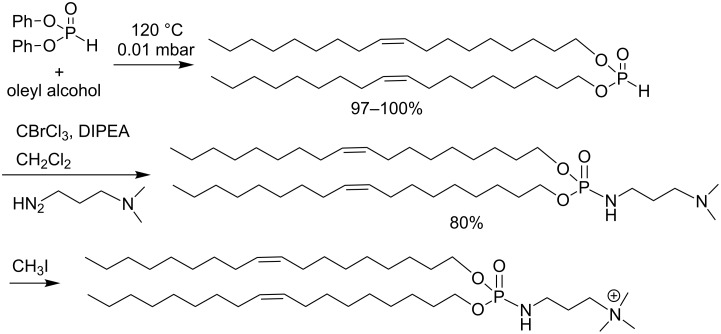
Synthesis of lipophosphite (bisoleyl phosphite) and cationic lipophosphoramidate with an AT reaction (adapted from [119]).

Cationic lipids are a promising class of compounds with the capacity to carry nucleic acids (DNA, RNA) for both in vitro and in vivo transfection assays. For the synthesis of cationic lipids, simple, efficient, modular procedures must be developed because the current applications require multifunctional carrying systems. Indeed, many nano-carriers are designed to respond to a specific stimulus (pH, red/ox, light) to trigger the drug release. To achieve the synthesis of polyfunctional amphiphilic compounds, the synthetic scheme must therefore be efficient, flexible and versatile. The AT reaction, which was used as a key step for the synthesis of neutral or cationic lipids, matches several of these properties. As a first example, cationic lipophosphoramidates can be produced in a three-step sequence that includes an AT reaction (Scheme 36). First, dioleyl phosphite can be readily prepared by a transesterification-like reaction between diphenyl phosphite and oleylalcohol. This reaction tolerates a large panel of lipid alcohol. These phosphite intermediates can be produced on a large scale (more than 50 g) and can be stored for a long period of time without any degradation in contrast to dialkyl chlorophosphate (another possible intermediate to produce amphiphilic compounds), which are water sensitive. In a second step, these phosphites can be engaged in the AT reaction as illustrated in Scheme 36. Bisoleyl phosphite was reacted with dimethylaminopropylamine to produce the expected phosphoramidate in good yield (80%) [119]. The reaction is carried out by adding DIPEA to a mixture of dioleyl phosphite, the nucleophilic amine and CBrCl_3_ in anhydrous dichloromethane. This addition was conducted at 5 °C followed by stirring the solution at 5 °C for 1 h. The concentration and extraction with a low polar solvent (hexane, diethyl ether), produced the expected phosphoramidate in almost quantitative yield. Purification was then achieved by column chromatography. In the last step, the cationic lipid was produced by reacting the tertiary amine functional group with iodomethane. Again, a simple washing (e.g., Et_2_O/water) produced the expected cationic phosphoramidate with good purity and in good yield (>80%).

**Scheme 37:**
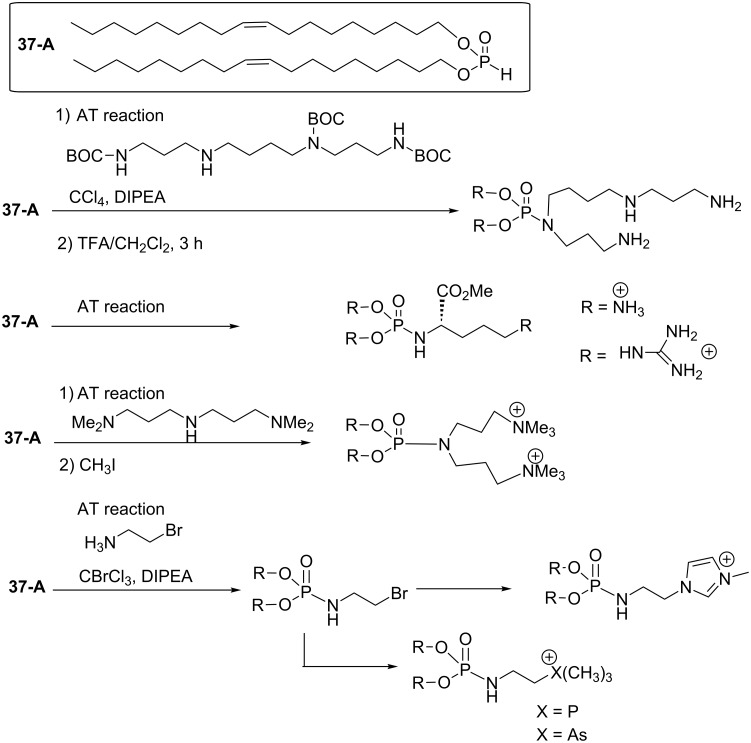
Use of AT reactions to produce cationic lipids characterized by a trimethylphosphonium, trimethylarsonium, guanidinium and methylimidazolium polar head.

In this sequence (Scheme 36) the AT reaction occupies a central place and with some adaptations a series of cationic lipids were synthesized, each of which characterized by different polar heads. These cationic lipids interact with nucleic acids mainly by electrostatic interactions, while the hydrophobic domains help to produce supramolecular aggregates (lipoplexes). Ideally, these aggregates must be as strong as possible to protect and to compact nucleic acids when these lipoplexes are localized in the systemic circulation (in vivo experiments) or in the supernatant media (in vitro experiments). However, after cell internalization, presumably by endocytosis processes, these lipoplexes must be as fragile as possible in order to escape lysosomal degradation. Consequently, the stability of the lipoplexes must be carefully tuned. The nature of the cationic polar head is one of the molecular features of the cationic lipids which directly influence the stability of lipoplexes by acting on the strength of the ionic interactions with the anion charge of the phosphate groups from the nucleic acids. The replacement of trimethylammonium with either trimethylphosphonium or trimethylarsonium offers better transfection efficacies probably owing to a better compromise between the stability and the instability of lipoplexes [120]. The synthesis of trimethylarsonium-based phosphoramidate was also achieved by using the AT reaction which proved to be an efficient tool to link the lipid part with the polar head region of the cationic lipid as exemplified in Scheme 37. With the same synthetic scheme, guanidinum-based lipophosphoramidates [121] obtained from natural amino acids, methylimidazolium [122], spermine-based amphiphile [123], dicationic lipophosphoramides [119] and arsonium or phosphonium [120] cationic lipids were synthesized.

**Scheme 38:**
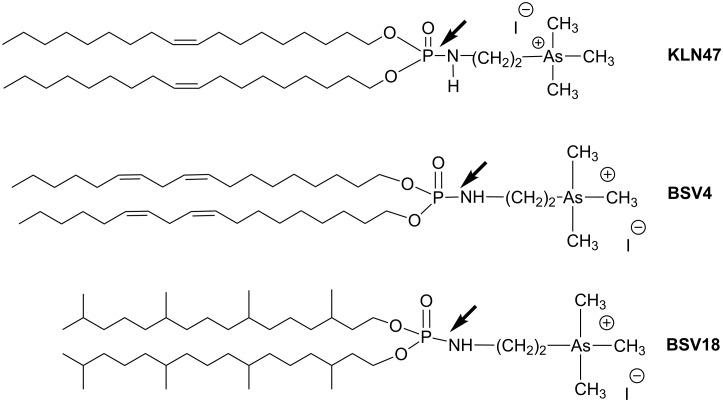
Cationic lipid synthesized by the AT reaction illustrating the variation of the structure of the lipid domain. The arrows indicate the bond formed by the AT reaction.

The lipid domain of lipophosphoramidates also influences the transfection process by acting on the physic-chemical properties of the supramolecular aggregates. Indeed, it was observed that bisphytanyl derivative BSV18 was particularly efficient for in vivo experiments. Presumably, this efficacy is based on the formation of an inverted hexagonal phase [124]. Once more, the AT reaction is a versatile reaction that allows for the production of a large panel of cationic lipids with unsaturated (e.g., KLN47 [125–126]), polyunsaturated (e.g., BSV4 [127]) or substituted alkyl chains (BSV18 [124]) as shown in Scheme 38.

**Scheme 39:**
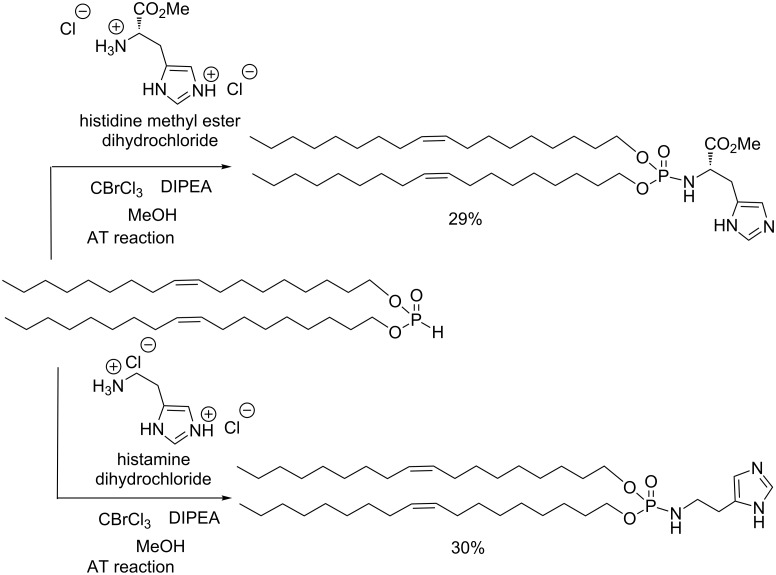
Helper lipids for nucleic acid delivery synthesized with the AT reaction (adapted from [130]).

In association with cationic lipids, helper lipids are frequently added to liposomal solutions with the aim to enhance transfection efficacies. DOPE (1,2-dioleyl-*sn*-glycero-3-phosphoethanolamine) is a natural phospholipid that was frequently employed as a helper lipid, since it favors the formation of a hexagonal phase at a pH of 6 [128–129]. In addition to natural helper lipids synthetic co-lipids were also developed. Neutral lipophosphoramidates were synthesized by AT reactions (Scheme 39) [130]. These helper lipids were obtained in a one-step reaction with 29–30% yield from dioleyl phosphite. The low yield was explained by the low solubility of the amine, which imposed the use of methanol as a solvent. Consequently, methanol acts as a second nucleophilic agent competing with the amine (histidine methyl ester or histamine). These yields can be improved (up to 50%) by reducing the volume of methanol used as a solvent in this reaction [131]. Interestingly, the liposomal solutions which incorporated the histamine-based helper lipid were also efficiently used to prepare lipopolyplexes (association of cationic lipid, cationic polymers and nucleic acid) employed for pDNA [132], RNA [133] and siRNA delivery [134].

**Scheme 40:**
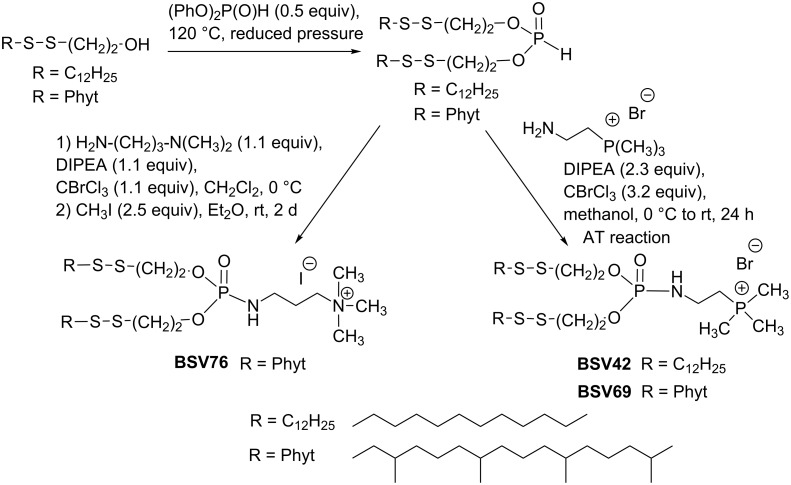
AT reaction used to produce red/ox-sensitive cationic lipids (adapted from [135]).

The question of the destabilization of lipoplexes after cell internalization can also be addressed by designing red/ox-sensitive cationic lipids. Accordingly, the incorporation of a disulfide bond, which can be cleaved by reducing agents naturally present in cytosol (glutathione), can induce a destabilization of the supramolecular aggregates. The AT reaction was also used for the synthesis of such red/ox-sensitive amphiphiles as illustrated in Scheme 40 [135]. The synthesis of lipophosphites incorporating two disulfide moieties was a key step. These phosphites were then engaged in the AT reaction to produce ammonium (BSV76) or phosphonium (BSV42 and BSV69) red/ox-sensitive cationic lipids in one or two steps. It was subsequently shown that lipoplexes prepared from these cationic lipids were destabilized in the presence of a reducing agent presumably caused by the breakdown of the S–S bond.

**Scheme 41:**
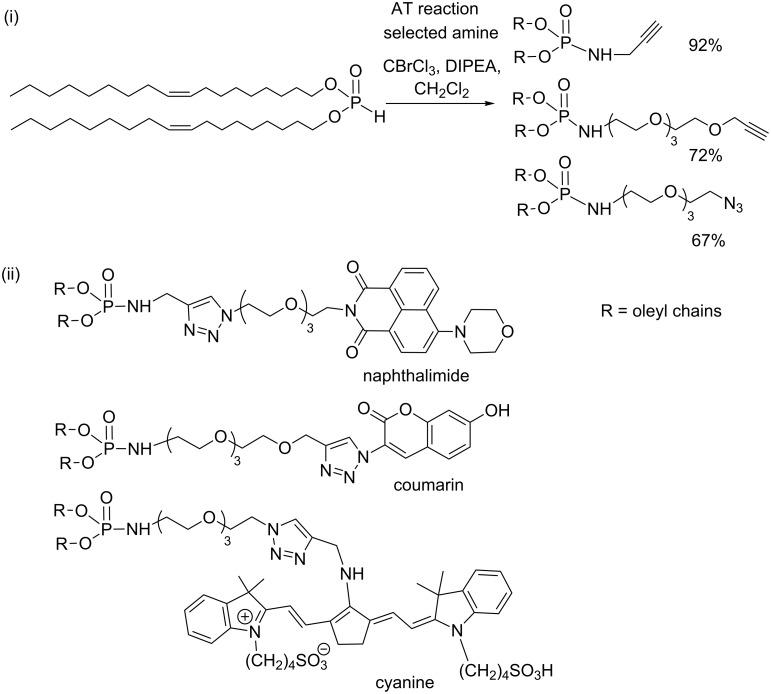
Alkyne and azide-functionalized phosphoramidate synthesized by AT reactions,(i); illustration of some fluorescent lipids synthesised from these intermediates, (ii) (adapted from [136]).

The introduction of a lipid domain on a molecule exhibiting specific properties (e.g., fluorescent or targeting group) is another goal needed to design tools for vectorization purposes. The use of click reactions for the production of polyfunctional amphiphiles (e.g., Huisgen cycloaddition) is very attractive. The combination of the AT reaction and click reaction (CuAAC) was reported to produce fluorescent lipids. The ‘clickable’ lipids (N_3_ or alkyne-functionalized phosphoramidates) were obtained by an AT reaction (Scheme 41-i). These intermediates were isolated with moderate to good yields (67–92%) at a 1 g scale after a purification step on silica gel [136]. These intermediates were then engaged in copper-catalyzed Huisgen cycloaddition to produce efficiently a series of fluorescent lipids like those shown in Scheme 41-ii.

**Scheme 42:**
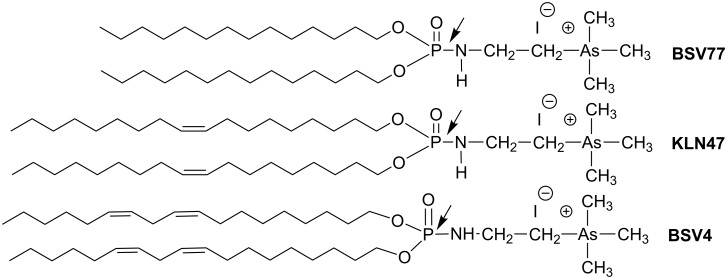
Cationic lipids exhibiting bactericidal action – arrows indicate the bond formed by the AT reaction (adapted from [137]).

Recently, Le Gall et al. [137] have reported that some lipophosphoramidates synthesized by AT reactions exhibited a remarkable bactericidal effect even on clinically relevant strains (*S. aureus* N315). Interestingly, the bactericidal effect was independent of the resistance profile of bacteria (e.g., MRSA). In a structure–activity study it has been shown that the presence of a trimethylarsonium polar head combined with a lipid domain were two structural features deeply influencing the bactericidal efficacies. The most efficient bactericidal agents were BSV77 and BSV4 (Scheme 42). Moreover, it was shown that lipoplexes, formed by the association of pDNA with BSV4, kept its bactericidal action. Additional experiments demonstrated that BSV4-based lipoplexes were able to simultaneously have a toxic effect on bacteria (bactericidal action) and a transfection capability for eukaryotic cells [137].

**Scheme 43:**
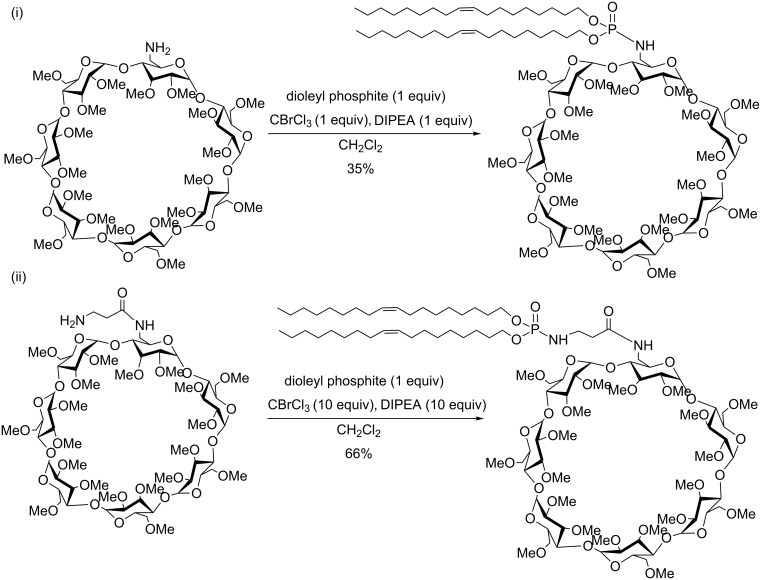
β-Cyclodextrin-based lipophosphoramidates (adapted from [138]).

β-Cyclodextrin (β-CD) is another molecular platform that was chemically modified with a lipid moiety introduced by an AT reaction [138]. As exemplified in Scheme 43, Djedaïni-Pilard et al. reported the use of the AT reaction to introduce a lipophosphoramidate fragment on a permethylated β-cyclodextrin possessing one primary amine (Scheme 43-i). After purification on silica gel, the expected lipophosphoramidate was isolated with 35% yield. The use of a spacer placed between the lipophosphoramide and the β-CD moiety (Scheme 43-ii) produced another permethylated β-CD with a better yield (66%). It is noteworthy that for this last reaction, an excess of CBrCl_3_ and DIPEA was used, which might explain the better yield. Finally, the same reaction achieved on non-methylated β-CD produced the lipophosphoramidate with very low yield (4%). It is probable that the alcohol functions and the residual water molecules compete as nucleophilic species in the AT reaction.

**Scheme 44:**
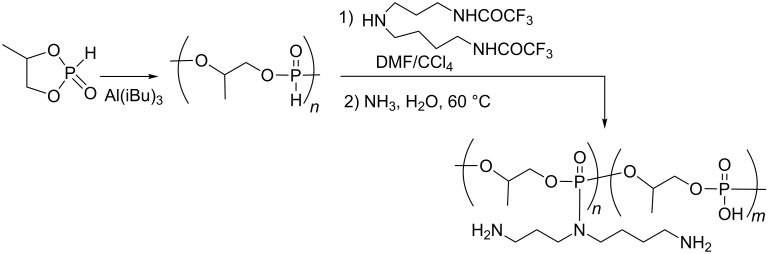
Polyphosphate functionalized by an AT reaction (adapted from [139]).

The AT reaction was also employed for the functionalization of polymeric materials that were subsequently used as a gene carrier. A polyphosphite was synthesised by ring-opening polymerization of 4-methyl-2-oxo-2-hydro-1,3,2-dioxaphopsholane with triisopropylaluminium (Scheme 44) [139]. Then, the AT reaction was carried out in a mixture of DMF/CCl_4_ with doubly protected spermidine (protection with trifluoroacetamide groups) as a nucleophile to produce a polyphosphoramidate. The deprotection of the primary amine with NH_3_ yielded the polyphosphoramidate branched with polyamine, which was subsequently used as a gene carrier. The functionalization of the polyphosphite with the AT reaction was compatible with a large panel of primary and secondary amines [140]. The post-functionalization of this polyphosphoramidate with monosaccharide or disaccharide groups has also been reported as a better transfection agent, especially for hepatocytes [141].

**Scheme 45:**
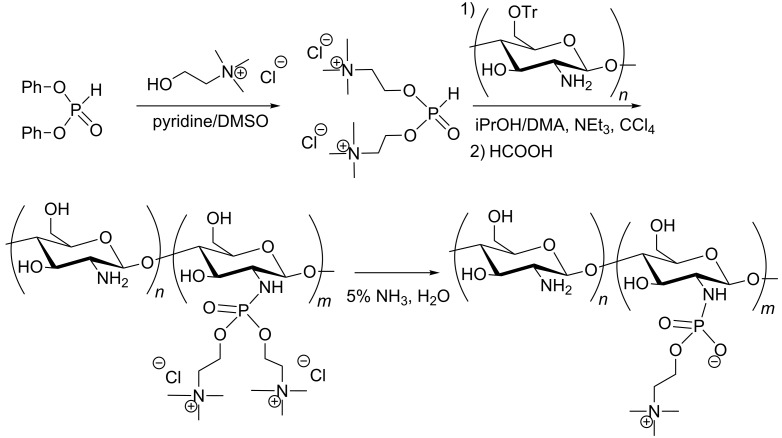
Synthesis of zwitterionic phosphocholine-bound chitosan (adapted from [142]).

Chitosan is another type of polymer functionalized with an AT reaction as shown in Scheme 45 [142]. The primary alcohol functions of chitosan were first protected with a trityl group. Then, the protected chitosan was engaged in an AT reaction that used a mixture of solvent (isopropanol/DMA), triethylamine as a base and CCl_4_ as a halogenating agent. The deprotection of the trityl group under basic conditions (NH_3_, H_2_O) led to the loss of one choline moiety.

**Scheme 46:**
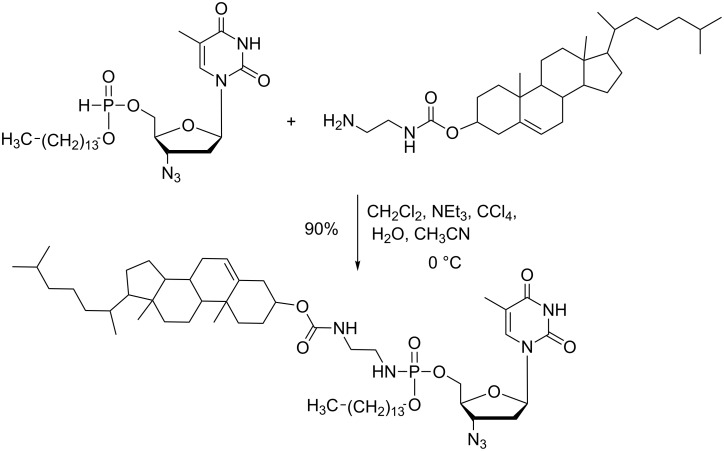
Synthesis of AZT-based prodrug via an AT reaction (adapted from [143]).

The AT reaction was also used to produce prodrugs as exemplified by the works of Zhao et al (Scheme 46), who reported the synthesis of phosphoramidates that included two lipid chains and one AZT moiety [143]. All phosphoramidates produced by this synthetic scheme exhibited high anti-HIV activities. The AT reaction can also be used to produce nucleoside phosphoramidate monoester [144] or thiophosphoramidates-based dinucleotides [77]. Other prodrugs were prepared by functionalization of polymers via a phosphoramidate tether. Yang et al. have reported the synthesis of chitosan functionalized with d4T (stavudine), a nucleoside reverse transcriptase inhibitor [145–146].

## Conclusion

The discovery of the reaction of dialkyl phosphite with amine in the presence of a base and CCl_4_ by Atherton, Openshaw and Todd in 1945 opened the way to a series of studies, which were initially focused on the investigation of the mechanism of this reaction. The most likely mechanism produced chloro- or bromophosphate as intermediate reactive species. This intermediate reacts in situ with a nucleophile in the presence of a base, which is involved to trap hydrochoride or hydrobromide, the byproducts of the AT reaction. The synthetic conditions were improved by employing stoichiometric quantities of CCl_4_ or CBCl_3_ instead of using them as a solvent. AT reactions can be used by chemists as a tool to activate phenol or amine functional groups, which may subsequently be engaged in reactions such as cyclization, reduction, cross coupling, the production of organometallic species or for the synthesis of arylphosphonates. Beside these synthetic applications, phosphoramidates or phosphates produced by AT reactions can be used for a variety of applications including organocatalysis, improvement of the fire resistance of polymers, prodrugs, and vectorization purposes. Among the recent published works, we would like to point out two promising domains, in which the AT reaction seems exceptionally attractive and worthwhile to be studied in depth. First, the current developments of vectorization systems aiming at being applied in the realm of personalized medicine require the synthesis of amphiphilic derivatives with several functionalities, e.g., fluorescent moieties, targeting groups, and PEG fragments, to produce stealthy nanoparticles. The accessibility of phosphite with two lipid chains renders the AT reaction very attractive for the incorporation of hydrophobic domains and thus provides a synthetic path to functionalized amphiphilic compounds. The second domain concerns the activation of small molecules like CO_2_ as recently illustrated by Y. F. Zhao et al. In these works, hydrospirophosphorane reacts with CO_2_ and a secondary amine to produce a phosphorylated carbamate derivative. Currently, the hydrospirophosphorane is used stoichiometrically. The development of a catalytic system characterized by hydrospirophosphorane or analogues acting as organocatalyst represents an alternative to organometallic catalysis in the field of CO_2_-based chemistry.

## References

[1] Atherton F R, Openshaw H T, Todd A R (1945). J Chem Soc.

[2] Atherton F R, Todd A R (1947). J Chem Soc.

[3] Hasse G (1877). Ber Dtsch Chem Ges.

[4] Lippmann E, Fleissner F (1886). Ber Dtsch Chem Ges.

[5] Fuson R C, Bull B A (1934). Chem Rev.

[6] Steinberg G M (1950). J Org Chem.

[7] Kamai G (1947). Compt Rend Acad Sci USSR.

[8] Kamai G (1948). J Gen Chem USSR.

[9] Krutikov V I, Erkin A V, Krutikova V V (2012). Russ J Gen Chem.

[10] Kong A, Engel R (1985). Bull Chem Soc Jpn.

[11] Cheymol J (1959). C R Chim.

[12] Cheymol J (1960). C R Chim.

[13] Troev K, Kirilov E M G, Roundhill D M (1990). Bull Chem Soc Jpn.

[14] Philippot E, Lindqvist O (1970). Acta Chem Scand.

[15] Emsley J, Lucas J, Parker R J, Ovrill R E (1983). Polyhedron.

[16] Georgiev E, Roundhill D M, Troev K (1992). Inorg Chem.

[17] Georgiev E M, Kaneti J, Troev K, Roundhill D M (1993). J Am Chem Soc.

[18] Kuiper J M, Hulst R, Engberts J B F N (2003). Synthesis.

[19] Antczak M I, Montchamp J-L (2008). Org Lett.

[20] Villemin D, Siméon F, Decreus H, Jaffrès P-A (1998). Phosphorus, Sulfur Silicon Relat Elem.

[21] Nilsson J, Kraszewski A, Strawinski J (2001). J Chem Soc, Perkin Trans 2.

[22] Wagner S, Rakotomalala M, Bykov Y, Walter O, Döring M (2012). Heteroat Chem.

[23] Hulst R, Feringa B L, de Vries N K (1992). Angew Chem, Int Ed Engl.

[24] Ji G-J, Xue C-B, Zeng J-N, Li L-P, Chai W-G, Zhao Y-F (1988). Synthesis.

[25] Hulst R, Kellogg R M, Feringa B L (1995). Recl Trav Chim Pays-Bas.

[26] Hulst R, Zijlstra R W J, Feringa B L, De Vries N K, ten Hoeve W, Wynberg H (1993). Tetrahedron Lett.

[27] Hulst R, Zijlstra R W J, de Vries N K, Feringa B L (1994). Tetrahedron: Asymmetry.

[28] Corriu R J P, Lanneau G F, Leclercq D (1986). Tetrahedron.

[29] Reiff L P, Aaron H S (1970). J Am Chem Soc.

[30] Stec W, Mikolajczyk M (1973). Tetrahedron.

[31] Zhou Y, Wang G, Saga Y, Shen R, Goto M, Zhao Y, Han L-B (2010). J Org Chem.

[32] Wang G, Shen R, Xu Q, Goto M, Zhao Y, Han L-B (2010). J Org Chem.

[33] Xiong B, Zhou Y, Zhao C, Goto M, Yin S-F, Han L-B (2013). Tetrahedron.

[34] Cao S, Guo Y, Wang J, Qi L, Gao P, Zhao H, Zhao Y (2012). Tetrahedron Lett.

[35] Kutyrev A A, Moskva V V, Alparova M V (1984). Zh Obshch Khim.

[36] Peng G, Du Y, Wei Y, Tang J, Peng A-Y, Rao L (2011). Org Biomol Chem.

[37] Mielniczak G, Bopusiński A (2003). Synth Commun.

[38] Lukanov L K, Venkov A P, Mollov N M (1985). Synthesis.

[39] Szardenings A K, Gordeev M F, Patel D V (1996). Tetrahedron Lett.

[40] Silverberg L J, Dillon J L, Vemishetti P (1996). Tetrahedron Lett.

[41] Mills S J, Dozol H, Vandeput F, Backers K, Woodman T, Erneux C, Spiess B, Potter B V L (2006). ChemBioChem.

[42] Lukanov L K, Venkov A P, Mollov N M (1986). Synth Commun.

[43] Dumitrascu A, Howell B A (2012). Polym Degrad Stab.

[44] Prokof’eva A F, Sapozhnikova Z Z, Pokrovskaya L A, Volkova V N, Negrebetskii V V, Golovkin G V, Mel’nikov N N (1988). Zh Obshch Khim.

[45] Zwierzak A, Sulewska A (1976). Synthesis.

[46] Koziara A, Turski K, Zwierzak A (1986). Synthesis.

[47] Chen G S, Wilbur J K, Barnes C L, Glaser R (1995). J Chem Soc, Perkin Trans 2.

[48] Verner J, Potacek M (2006). Molecules.

[49] Matthews H, Ranson M, Tyndall J D A, Kelso M J (2011). Bioorg Med Chem Lett.

[50] Wadsworth W S, Emmons W D (1964). J Org Chem.

[51] Zwierzak A, Brylikowska J (1975). Synthesis.

[52] Jones S, Selitsianos D, Thompson K J, Toms S M (2003). J Org Chem.

[53] Edsall A B, Agoston G E, Treston A M, Plum S M, McClanahan R H, Lu T-S, Song W, Cushman M (2007). J Med Chem.

[54] Bonnaventure I, Charrette A B (2008). J Org Chem.

[55] Selikhov A N, Malyshera Y B, Nyuchev A V, Sitnikov N S, Sharonova E A, Shavyrin A S, Combes S, Fedorov A Yu (2011). Russ Chem Bull.

[56] Jayasundera K P, Watson A J, Taylor C M (2005). Tetrahedron Lett.

[57] Ilia G, Bila S, Popa A, Illiescu S, Macarie L, Plescu N (2005). Rev Chim.

[58] Ding Y, Huang X (2001). Synth Commun.

[59] Shioiri T, Ninomiya S, Yamada S (1972). J Am Chem Soc.

[60] Cai Q, He G, Ma D (2006). J Org Chem.

[61] Hamada Y, Shioiri T (2005). Chem Rev.

[62] Yamada S, Kasai Y, Shioiri T (1973). Tetrahedron Lett.

[63] Yokoyama Y, Shioiri T, Yamada S (1977). Chem Pharm Bull.

[64] Thompson A S, Humphrey G R, DeMarco A H, Mathre D J, Grabowski E J J (1993). J Org Chem.

[65] Cremlyn R J W (1973). Aust J Chem.

[66] Shi E, Pei C (2003). Phosphorus, Sulfur Silicon Relat Elem.

[67] Shi E, Pei C (2004). Synthesis.

[68] Shi E, Pei C (2005). Synth Commun.

[69] Cao S, Gao P, Guo Y, Zhao H, Wang J, Liu Y, Zhao Y (2013). J Org Chem.

[70] Neisius M, Liang S, Mispreuve H, Gaan S (2013). Ind Eng Chem Res.

[71] Ortial S, Fisher H C, Montchamp J-L (2013). J Org Chem.

[72] Bondarenko N A, Kharlamov A V, Vendilo A G (2009). Russ Chem Bull.

[73] Gusarova N K, Volkov P A, Ivanova N I, Oparina L A, Kolyvanov N A, Vysotskaya O V, Larina L I, Trofimov B A (2012). Synthesis.

[74] Ivanova N I, Volkov P A, Larina L I, Gusarova N K, Trofimov B A (2012). Chem Heterocycl Compd.

[75] Gusarova N K, Volkov P A, Ivanova N I, Larina L I, Trofimov B A (2011). Synthesis.

[76] Gusarova N K, Volkov P A, Ivanova N I, Larina L I, Trofimov B A (2012). Heteroat Chem.

[77] Lin C, Fu H, Tu G, Zhao Y (2003). Synthesis.

[78] Bartoszewicz A, Kalek M, Stawinski J (2008). J Org Chem.

[79] Minaeva L I, Patrikeeva L S, Kabachnik M M, Beletskaya I P (2010). Russ J Org Chem.

[80] Fraser J, Wilson L J, Blundell R K, Hayes C J (2013). Chem Commun.

[81] Kenner G W, Williams N R (1955). J Chem Soc.

[82] Duclos R I, Lu D, Guo J, Makriyannis A (2008). Tetrahedron Lett.

[83] Faldt A, Krebs F C, Thorup N (1997). J Chem Soc, Perkin Trans 2.

[84] Lusch M J, Woller K R, Keller A M, Turk M C (2005). Synthesis.

[85] Yang T, Lin C, Fu H, Jiang Y, Zhao Y (2005). Org Lett.

[86] Chopa A B, Lockhart M T, Dorn V B (2002). Organometallics.

[87] Chopa A B, Lockhart M T, Silbestri G (2000). Organometallics.

[88] Dorn V B, Silbestri G F, Lockhart M T, Chopa A B, Pierini A B (2013). New J Chem.

[89] Chen H, Huang Z, Hu X, Tang G, Xu P, Zhao Y, Cheng C-H (2011). J Org Chem.

[90] Yoshikai N, Matsuda H, Nakamura E (2009). J Am Chem Soc.

[91] Dhawan B, Redmore D (1984). J Org Chem.

[92] Melvin L S (1981). Tetrahedron Lett.

[93] Marie S, Lutz M, Spek A L, Klein Gebbink R J M, van Koten G, Kervarec N, Michaud F, Salaün J-Y, Jaffrès P-A (2009). J Organomet Chem.

[94] Taylor C M, Watson A J (2004). Curr Org Chem.

[95] Huang J-H, Yang L-M (2011). Org Lett.

[96] Flowers R A, Xu X, Timmons C, Li G (2004). Eur J Org Chem.

[97] Beutner G L, Denmark S E (2013). Top Organomet Chem.

[98] Denmark S E, Su X, Nishigaichi Y, Coe D-M, Wong K-T, Winter S B D, Choi J Y (1999). J Org Chem.

[99] Mummy F, Haag R (2012). Synlett.

[100] Ashby J, Styles A J, Anderson D (1977). Br J Cancer.

[101] Denmark S E, Winter S B D, Su X, Wong K-T (1996). J Am Chem Soc.

[102] Denmark S E, Bui T (2004). Proc Natl Acad Sci U S A.

[103] Ding M, Zhou F, Liu Y-L, Wang C-H, Zhao X-L, Zhou J (2011). Chem Sci.

[104] Nakashima D, Yamamoto H (2006). J Am Chem Soc.

[105] Wang S-G, Han L, Zeng M, Sun F-L, Zhang W, You S-L (2012). Org Biomol Chem.

[106] Hulst R, Heres H, Peper N C M W, Kellogg R M (1996). Tetrahedron: Asymmetry.

[107] Hulst R, Heres H, Fitzpatrick K, Peper N C M W, Kellogg R M (1996). Tetrahedron: Asymmetry.

[108] Bravo-Altamirano L, Coudray L, Deal E L, Montchamp J-L (2010). Org Biomol Chem.

[109] Rakotomalala M, Wagner S, Döring M (2010). Materials.

[110] Granzow A (1978). Acc Chem Res.

[111] Jones D M, Noone T M (1962). J Appl Chem.

[112] Wilson B N, Gordon I, Hindersinn R R (1974). Ind Eng Chem Prod Res Dev.

[113] Gaan S, Rupper P, Salimova V, Heuberger M, Rabe S, Vogel F (2009). Polym Degrad Stab.

[114] Rupper P, Gaan S, Salimova V, Heuberger M (2010). J Anal Appl Pyrolysis.

[115] Nguyen T-M, Chang S, Condon B, Slopek R, Graves E, Yoshioka-Tarver M (2013). Ind Eng Chem Res.

[116] Gaan S, Mauclaire L, Rupper P, Salimova V, Tran T-T, Heuberger M (2011). J Anal Appl Pyrolysis.

[117] Bykov Y, Wagner S, Walter O, Döring M, Fisher O, Pospiech D, Köppl T, Altstädt V (2012). Heteroat Chem.

[118] Zang L, Wagner S, Ciesielski M, Müller P, Döring M (2011). Polym Adv Technol.

[119] Mével M, Montier T, Lamarche F, Delépine P, Le Gall T, Yaouanc J-J, Jaffrès P-A, Cartier D, Lehn P, Clément J-C (2007). Bioconjugate Chem.

[120] Berchel M, Le Gall T, Couthon-Gourvès H, Haelters J-P, Montier T, Midoux P, Lehn P, Jaffrès P-A (2012). Biochimie.

[121] Mével M, Breuzard G, Yaouanc J-J, Clément J-C, Lehn P, Pichon C, Jaffrès P-A, Midoux P (2008). ChemBioChem.

[122] Midoux P, Pichon C, Yaouanc J- J, Jaffrès P-A (2009). Br J Pharmacol.

[123] Lamarche F, Mével M, Montier T, Burel-Deschamps L, Giamarchi P, Tripier R, Delépine P, Le Gall T, Cartier D, Lehn P (2007). Bioconjugate Chem.

[124] Lindberg M F, Carmoy N, Le Gall T, Fraix A, Berchel M, Lorilleux C, Couthon-Gourvès H, Bellaud P, Fautrel A, Jaffrès P-A (2012). Biomaterials.

[125] Picquet E, Le Ny K, Delépine P, Montier T, Yaouanc J-J, Cartier D, des Abbayes H, Férec C, Clément J-C (2005). Bioconjugate Chem.

[126] Laurent V, Fraix A, Montier T, Cammas-Marion S, Ribault C, Benvegnu T, Jaffrès P-A, Loyer P (2010). Biotechnol J.

[127] Le Gall T, Loizeau D, Picquet E, Carmoy N, Yaouanc J-J, Burel-Deschamps L, Delépine P, Giamarchi P, Jaffrès P-A, Lehn P (2010). J Med Chem.

[128] Safinya C R (2001). Curr Opin Struct Biol.

[129] Koynova R, Wang L, MacDonald R C (2006). Proc Natl Acad Sci U S A.

[130] Mével M, Neveu C, Gonçalves C, Yaouanc J-J, Pichon C, Jaffrès P-A, Midoux P (2008). Chem Commun.

[R131] 131Unpublished results.

[132] Perche F, Gosset D, Mével M, Miramon M-L, Yaouanc J-J, Pichon C, Benvegnu T, Jaffrès P-A, Midoux P (2011). J Drug Targeting.

[133] Perche F, Benvegnu T, Berchel M, Lebegue L, Pichon C, Jaffrès P-A, Midoux P (2011). Nanomedicine.

[134] Gonçalves C, Berchel M, Gosselin M-P, Malard V, Cheradame H, Jaffrès P-A, Guégan P, Pichon C, Midoux P (2014). Int J Pharm.

[135] Fraix A, Le Gall T, Berchel M, Denis C, Lehn P, Montier T, Jaffrès P-A (2013). Org Biomol Chem.

[136] Berchel M, Haelters J-P, Couthon-Gourvès H, Deschamps L, Midoux P, Lehn P, Jaffrès P-A (2011). Eur J Org Chem.

[137] Le Gall T, Berchel M, Le Hir S, Fraix A, Salaün J Y, Férec C, Lehn P, Jaffrès P-A, Montier T (2013). Adv Healthcare Mater.

[138] Gervaise C, Bonnet V, Wattraint O, Aubry F, Sarazin C, Jaffrès P-A, Djedaïni-Pilard F (2012). Biochimie.

[139] Wang J, Zhang P-C, Lu H-F, Ma N, Wang S, Mao H-Q, Leong K W (2002). J Controlled Release.

[140] Zhang P-C, Wang J, Leong K W, Mao H-Q (2005). Biomacromolecules.

[141] Zhang X-Q, Wang X-L, Zhang P-C, Liu Z-L, Zhuo R-X, Mao H-Q, Leong K W (2005). J Controlled Release.

[142] Zeng R, Fu H, Zhao Y (2006). Macromol Rapid Commun.

[143] Jin P, Liu K, Ji S, Ju Y, Zhao Y (2007). Synthesis.

[144] Zhu J, Fu H, Jiang Y, Zhao Y (2005). Synlett.

[145] Yang L, Zeng R, Li C, Li G, Qiao R, Hu L, Li Z (2009). Bioorg Med Chem Lett.

[146] Yang L, Chen L, Zeng R, Li C, Qiao R, Hu L, Li Z (2010). Bioorg Med Chem.

